# Older adults’ evaluations of smart home control screen designs: Appearance dimensions and behavioral intentions in a multi-stage study

**DOI:** 10.1371/journal.pone.0358862

**Published:** 2026-10-08

**Authors:** Liangming Jia, Qisen Zhang, Weimin Zhai, Zhongzhen Lin, Shasha Li

**Affiliations:** 1 School of Media and Arts, Wuxi University, Wuxi, China; 2 Undergraduate Academic Affairs Office, Wuxi Open University, Wuxi, China; 3 Department of Industrial Design, Shenzhen Technology University, Shenzhen, China; 4 Department of Industrial Design, Guangdong University of Technology, Guangzhou, China; 5 College of Engineering, South China Agricultural University, Guangzhou, China; Philadelphia University, JORDAN

## Abstract

In age-friendly home environments, control screens provide a point of contact between users and smart home systems, and their appearance may shape initial evaluations of these systems. This exploratory study used a multi-stage design to investigate older adults’ appearance evaluations and behavioral-intention patterns across different screen designs. Similarity judgments provided by 15 experts were examined using multidimensional scaling and cluster analysis, from which eight representative designs were selected from the original pool of 87 samples. Three independent groups of older adults took part, respectively, in dimension construction, comparative appearance evaluation, and behavioral-intention assessment. Exploratory principal component analysis identified four dimensions specific to the present study: *Sturdy and Reliable*, *Futuristic Technology*, *High Quality*, and *Simple and Efficient*. In the supplementary entropy-weighted TOPSIS analysis, the two largest data-derived weights were assigned to *Sturdy and Reliable* (31.3%) and *Simple and Efficient* (28.8%), and S73 ranked first in the composite appearance evaluation. Preference, willingness to use, and willingness to purchase differed across the eight designs in the Friedman tests (all p < 0.001). S56 had the highest ranking for preference and willingness to use, while S69 ranked highest for willingness to purchase. At the sample level, S73 and S56 performed comparatively well at both stages, although the composite appearance and behavioral-intention rankings did not fully coincide. Because the two sets of outcomes came from independent participant groups, comparisons across the two stages were descriptive only. These findings provide a basis for subsequent development and prototype testing of age-friendly interfaces.

## Introduction

Population aging is changing the demands placed on healthcare, residential environments, and everyday support for older adults worldwide. By the end of 2024, China had more than 300 million people aged 60 years or older, representing over 20% of the population, and this proportion continues to rise [1]. In response, national policies have placed greater emphasis on home- and community-based care services and their supporting infrastructure [2,3]. Smart home products form part of this age-friendly environment by supporting safety monitoring, health management, and daily activities [4]. Among these products, smart home control screens serve as important user–system interfaces through which older adults encounter, interpret, and evaluate smart home functions in everyday domestic settings.

As interfaces connecting users with smart home systems, smart home control screens contribute to how users perceive, understand, and interact with digital home environments. Previous research on smart home interfaces primarily examined interaction techniques, system performance, and operational efficiency. For example, studies have explored gesture-based control systems [5], speech-recognition-based smart environments [6], and the influence of interface layout and input modes on interaction efficiency [7]. An increasing number of studies have also considered age-related differences in interaction performance, suggesting that interface design can affect older users’ task completion, error rates, and perceived usability [8–10]. Together, these studies describe smart home interface use primarily in relation to interaction efficiency and age-related usability.

Appearance evaluation provides another useful perspective for understanding smart home control screen designs, because product appearance offers users early semantic and affective cues before actual interaction takes place. Users’ evaluations of product appearance may involve judgments related to form, materials, and color. Among these factors, appearance serves not only as the structural basis for functional implementation but also as a primary medium through which emotional and semantic attributes are conveyed. When material and color conditions are held constant, variations in form semantics alone can be associated with differences in users’ evaluative impressions [11]. Evidence from other visually oriented design and service contexts suggests that visual stimuli can shape attitudes and behavioral responses [12,13]. Several empirical studies have examined appearance-related and interaction-related characteristics of smart home or control interfaces from different perspectives. For example, Lin et al. [14] investigated how morphological attributes—such as corner type, border design (full-border versus borderless), and screen orientation (horizontal versus vertical)—influence users’ aesthetic preferences. Tory and Kincaid [15] compared touch, physical, and touch-plus-overlay parameter controls and reported differences in interaction performance and visual attention. Accordingly, control-interface appearance can be considered both as a formal attribute and as a source of evaluative impressions related to design alternatives.

Prior studies have examined smart home interfaces from the perspectives of interaction technique, interface layout, input mode, and age-related usability [5–10]. Extending this line of inquiry to appearance-oriented comparison, the present study adopted a multi-stage analytical framework to examine appearance evaluation dimensions, differences across representative smart home control screen samples, and sample-level comparisons between appearance evaluation outcomes and behavioral intention ratings obtained from a separate participant group. Specifically, this study addressed the following research questions:

(1) What appearance evaluation dimensions do older adults use when assessing smart home control screen designs?
(2) How do representative smart home control screen designs differ across these appearance evaluation dimensions?
(3) What similarities and differences are observed in sample-level patterns between appearance evaluation outcomes and behavioral intention ratings across the representative smart home control screen designs?

In this study, appearance evaluation and behavioral intention were examined in separate participant groups and at different analytical stages. The comparison of the two therefore focused on sample-level patterns across representative designs rather than on individual-level prediction. As a brief illustration of the application of the proposed framework, the representative design S73, characterized by a medium-sized 16:9 screen, a high screen-to-body ratio, and touch-based control, received mean ratings of 5.06–5.48 across the four appearance evaluation dimensions. The entropy-weighted technique for order preference by similarity to ideal solution (TOPSIS) integrated these ratings into a closeness coefficient of 0.778, corresponding to the highest composite ranking among the eight representative designs. In the separate behavioral-intention assessment, S73 also showed relatively favorable preference and willingness-to-use ratings. This example links design characteristics with dimension-specific appearance ratings, composite comparison, and sample-level behavioral-intention patterns.

The remainder of the paper follows the sequence of the multi-stage study. The next section explains the conceptual basis and analytical framework adopted in the study. The study design and procedures are presented next, followed by the findings from representative-sample screening, appearance-dimension construction, multidimensional synthesis, and behavioral-intention comparison. The Discussion examines the meaning and implications of the findings, while the final section presents the conclusions, limitations, and directions for further research.

## Conceptual and analytical framework

### Appearance evaluation and Kansei engineering

Kansei engineering uses descriptive vocabulary, most often adjectives, to elicit users’ affective and evaluative responses to product designs [16,17]. Beyond functional performance, the approach examines how users’ subjective impressions can be articulated, organized, and related to product characteristics in ways that are meaningful for design practice. Product-appearance and interface studies have applied Kansei engineering to visual and formal cues that may not be adequately reflected in conventional usability measures. From this perspective, appearance conveys semantic and emotional meaning rather than functioning only as a surface property [11]. Appearance-related cues, including form and proportion, may shape evaluative impressions, and specific formal attributes have been associated with differences in users’ aesthetic responses [14].

A Kansei engineering study generally involves selecting representative objects and defining their attributes [18], deriving evaluation dimensions from users’ affective judgments [19], and relating the resulting responses to design characteristics [19,20]. In this study, affective adjectives were used to organize older adults’ impressions into appearance dimensions that could subsequently be compared across the representative designs. Product-aesthetics research distinguishes aesthetic, semantic, and symbolic responses to visual form [21], whereas age-friendly technology research indicates that these responses may operate alongside perceived ease of use, prior experience, and other determinants of technology adoption [22,23].

### Representative samples and multidimensional scaling

Smart home control screens differ considerably in appearance, making it impractical for older adult participants to evaluate every available design. Selection based only on market share or sales rankings would also not necessarily retain sufficient morphological variation for an appearance-based comparison. Multidimensional scaling (MDS) was therefore applied to expert-derived dissimilarity judgments. The resulting lower-dimensional configuration represented relationships among the samples and provided the coordinates used for clustering and representative-sample selection [24–26].

MDS retained the relative ordering of the perceived dissimilarities, and clustering reduced this configuration to a manageable set of sufficiently distinct designs for the subsequent evaluations. This procedure was intended to retain variation within the assembled sample pool, rather than to identify products that are universally representative of smart home control screens.

### Appearance evaluation dimensions and exploratory principal component analysis

Many adjectives used to describe smart home control-screen appearance overlap in meaning and are closely interrelated. Expert screening can retain terms that are relevant to appearance, semantically clear, and understandable to older adult participants [18,19]. However, it cannot determine how the target group organizes these adjectives when evaluating the designs. We therefore examined empirically how the selected adjectives were grouped in older adults’ ratings when constructing the appearance dimensions [27,28]. The aim was to obtain a concise set of appearance dimensions for comparing the representative samples, rather than simply to shorten the descriptor list.

Exploratory principal component analysis (PCA) was therefore employed to examine the grouping structure underlying older adults’ evaluations of the selected descriptive adjectives [27,28]. Principal component extraction was followed by Varimax rotation with Kaiser normalization to obtain a clearer and more interpretable component structure. In this study, PCA served as the primary exploratory procedure for identifying appearance evaluation dimensions from the current dataset. These dimensions were empirically derived and interpretive groupings within the present research context, rather than definitive or universally generalizable psychological constructs. The suitability of the data for PCA was assessed using the Kaiser–Meyer–Olkin (KMO) measure and Bartlett’s test of sphericity [29], while component loadings were used to evaluate the strength of association between individual adjectives and the extracted components [27,29].

### Multidimensional synthesis using entropy-weighted TOPSIS

Appearance evaluation of smart home control screen designs involves multiple evaluative dimensions, and representative samples may receive different ratings across them. The study therefore used an integrative synthesis to compare the samples across dimensions. This procedure complemented rather than replaced the dimension-specific results.

Within this framework, entropy-weighted TOPSIS was used to synthesize ratings across the appearance evaluation dimensions [14,30–32]. The entropy weighting procedure was used to reflect the relative discriminatory contribution of each dimension within the present dataset [31]. TOPSIS then supported the comparative ranking of representative samples by considering their relative proximity to idealized evaluation profiles [32]. In this study, the resulting weights and rankings were used only for structured comparison within the current dataset and sample set. The principal mathematical notations used in the entropy-weighted TOPSIS procedure are summarized in S1 Table. The computational steps are described below.

Assume that there are *m* participants and *n* evaluation criteria. Following the standard entropy-weight procedures described in previous multi-criteria decision-making studies [31,32], the participant-by-criterion rating matrix was first constructed as shown in Equation (1):


$$\begin{array}{c} \text{X}=\left[x_{ij}\right]=[\begin{array}{cccc} x_{\text{11}} & x_{\text{12}} & \ldots & x_{\text{1n}}\\ x_{\text{21}} & x_{\text{22}} & \ldots & x_{\text{2n}}\\ : & : & : & :\\ x_{\text{m1}} & x_{\text{m2}} & \ldots & x_{\text{mn}} \end{array}] \end{array}$$ (1)

where $x_{ij}$ denotes the rating given by participant *i* on evaluation criterion *j*, with *i* = 1, ..., *m* and *j* = 1, ..., *n*. To proceed, the decision matrix must be normalized to ensure comparability across different criteria. The normalization process is performed as described in Equation (2):


$$\begin{array}{c} r_{ij}=\frac{x_{ij}}{\sum_{i=\text{1}}^{m}x_{ij}} \end{array}$$ (2)

Normalization involves calculating the ratio of each matrix value to the sum of all values in its corresponding column. The resulting normalized rating matrix is represented by $r_{ij}$. The entropy of the *j*-th criterion, denoted as $e_{j}$, is then calculated as shown in Equation (3):


$$\begin{array}{c} e_{j}=-h\sum_{i=\text{1}}^{m}r_{ij}lnr_{ij},\;i=\text{1},\text{2},\ldots ,m;j=\text{1},\text{2},\ldots ,n \end{array}$$ (3)

where h = 1/ln (*m*), and *m* represents the number of observational units. The next step is to calculate the criterion weight $w_{j}$, as shown in Equation (4):


$$\begin{array}{c} w_{j}=\frac{\text{1}-e_{j}}{\sum_{j=\text{1}}^{n}(\text{1}-e_{j})},j=\text{1},\text{2},\ldots ,n \end{array}$$ (4)

The quantity $d_{j}$ = 1-$e_{j}$ is referred to as the degree of diversification contained within each criterion [31]. A higher value of $d_{j}$ corresponds to a higher weight.

TOPSIS was then applied to combine the weighted dimension scores and produce an overall comparative ranking of the designs. For each design, TOPSIS compares its distance from the positive and negative ideal solutions; designs closer to the former and farther from the latter receive higher rankings [32]. The calculation proceeded as follows.

For the TOPSIS step, the sample-level decision matrix was normalized using Equation (5):


$$\begin{array}{c} \overset{―}{X_{ij}}=\frac{X_{ij}}{\sqrt{\sum_{i=\text{1}}^{m}X_{ij}^{\text{2}}}} \end{array}$$ (5)

Subsequently, the weighted normalized decision matrix is calculated using Equation (6):


$$\begin{array}{c} V_{ij}=w_{j}\times \overset{―}{X_{ij}} \end{array}$$ (6)

Each evaluation criterion has a corresponding weight ($w_{j}$), and the sum of the weights across all criteria equals 1, i.e., $\sum_{j=\text{1}}^{n}w_{j}=\text{1}$, ensuring a normalized weighting scheme.

Let B denote the set of benefit criteria and C denote the set of cost criteria. The positive ideal solution ($A^{*}$) and the negative ideal solution ($A^{-}$) are defined using Equations (7) and (8):


$$\begin{array}{c} A^{*}=\left\{\left({max}_{i}V_{ij}|j\in B\right),({min}_{i}V_{ij}|j\in C)|i=\text{1},\text{2},\ldots ,m\right\}=\left\{V_{\text{1}}^{*},V_{\text{2}}^{*},\ldots ,V_{j}^{*},\ldots ,V_{n}^{*}\right\} \end{array}$$ (7)

$$\begin{array}{c} A^{-}=\left\{\left({min}_{i}V_{ij}|j\in B\right),({max}_{i}V_{ij}|j\in C)|i=\text{1},\text{2},\ldots ,m\right\}=\left\{V_{\text{1}}^{-},V_{\text{2}}^{-},\ldots ,V_{j}^{-},\ldots ,V_{n}^{-}\right\} \end{array}$$ (8)

In this study, all appearance evaluation dimensions were treated as benefit criteria. The Euclidean distances from each alternative to the positive and negative ideal solutions were calculated using Equations (9) and (10), respectively:


$$\begin{array}{c} S_{i}^{*}={\left[\sum_{j=\text{1}}^{n}{(V_{ij}-V_{j}^{*})}^{\text{2}}\right]}^{\text{0}.\text{5}} \end{array}$$ (9)

$$\begin{array}{c} S_{i}^{-}={\left[\sum_{j=\text{1}}^{n}{(V_{ij}-V_{j}^{-})}^{\text{2}}\right]}^{\text{0}.\text{5}} \end{array}$$ (10)

Let $C_{i}^{*}$ represent the relative closeness of alternative $A_{i}$ to the positive ideal solution ($A^{*}$). The relative closeness coefficient was calculated using Equation (11):


$$\begin{array}{c} C_{i}^{*}=\frac{S_{i}^{-}}{S_{i}^{-}+S_{i}^{*}} \end{array}$$ (11)

The alternatives were then ranked according to the magnitude of $C_{i}^{*}$, with values closer to 1 indicating greater proximity to the positive ideal solution. This procedure was used as a comparative synthesis tool for representative samples across multiple appearance evaluation dimensions, rather than as evidence of universally generalizable importance weights or normative design priorities. To examine the sensitivity of the ranking to the weighting scheme, TOPSIS was additionally repeated using equal weights (0.25 for each dimension), and rank-order agreement with the entropy-weighted solution was assessed using Spearman’s rank correlation.

### Behavioral intention comparison and non-parametric analysis

Behavioral intentions were treated as comparative outcomes across representative smart home control screen samples, including preference, willingness to use, and willingness to purchase. In studies of product appearance evaluation and user-centered design, such behavioral responses are commonly examined through comparative judgments across alternative design options [33]. The purpose of this part of the analysis was therefore to examine whether different representative samples elicited different response tendencies among older adults, thereby providing a comparative perspective on how alternative designs were received. Behavioral intention was therefore analyzed as a comparative sample-based outcome, with emphasis on differences across representative samples rather than individual-level prediction or causal inference.

Because appearance evaluation and behavioral intention were examined in separate participant groups, the analysis focuses on structured comparisons of sample-level patterns across representative smart home control screen designs rather than individual-level associations between the two. Within the behavioral-intention stage, however, the same participants evaluated all eight representative samples, resulting in related repeated-measures data. Overall differences in preference, willingness to use, and willingness to purchase across the eight samples were therefore examined separately using Friedman tests [34,35]. When significant overall differences were identified, Nemenyi post hoc comparisons were conducted to examine pairwise differences in ranks [35]. Wilcoxon signed-rank tests were additionally performed as complementary pairwise analyses [34,35], with Holm adjustment applied separately within each behavioral intention outcome across all 28 pairwise comparisons among the eight samples [36].

The analytical techniques discussed above address different methodological tasks and involve distinct strengths and limitations. Table 1 summarizes their principal advantages and limitations in relation to appearance evaluation, sample reduction, dimension construction, multidimensional synthesis, and comparative analysis. In the present study, these techniques were therefore used in a complementary multi-stage framework rather than as interchangeable alternatives.

**Table 1:** Comparison of analytical methods relevant to the present multi-stage framework.

Analytical method	Primary purpose	Advantages	Limitations	Role in this study
Kansei engineering	Capturing affective and semantic responses to product appearance	Links subjective impressions with design attributes	Sensitive to adjective selection, stimuli, and target users	Basis for appearance evaluation
MDS	Mapping similarity relationships in reduced-dimensional space	Visualizes complex similarity structures	Depends on input dissimilarities and dimensional solution	Supported sample representation
Cluster analysis	Grouping similar objects	Enables structured sample reduction	Sensitive to cluster number, initialization, and sample structure	Selected representative designs
Exploratory PCA	Identifying interpretable component groupings among correlated descriptors	Reduces correlated descriptors to a smaller empirical structure	Components are sample- and context-dependent	Derived four appearance evaluation dimensions
Entropy weighting	Deriving data-driven criterion weights	Reduces reliance on subjective weighting	Reflects data variation rather than absolute importance	Determined dimension weights
TOPSIS	Ranking alternatives across multiple criteria	Provides an interpretable composite ranking	Sensitive to weighting and normalization choices	Compared representative designs
Friedman, Nemenyi, and Wilcoxon signed-rank tests	Comparing related ordinal ratings across multiple conditions	Accommodate repeated-measures data and support omnibus and pairwise comparisons	Rank-based comparisons do not establish causality	Compared behavioral intentions across representative designs

## Study design and procedures

The study was organized into four analytical phases, as illustrated in Fig 1. These phases corresponded to representative-sample screening, appearance-dimension construction, comparative appearance evaluation, and behavioral-intention comparison. The outputs of the representative-sample screening and appearance-dimension construction phases informed the later evaluation phases. Appearance evaluation and behavioral intention were assessed in separate older-adult groups and were compared only at the sample level.

**Fig 1:**
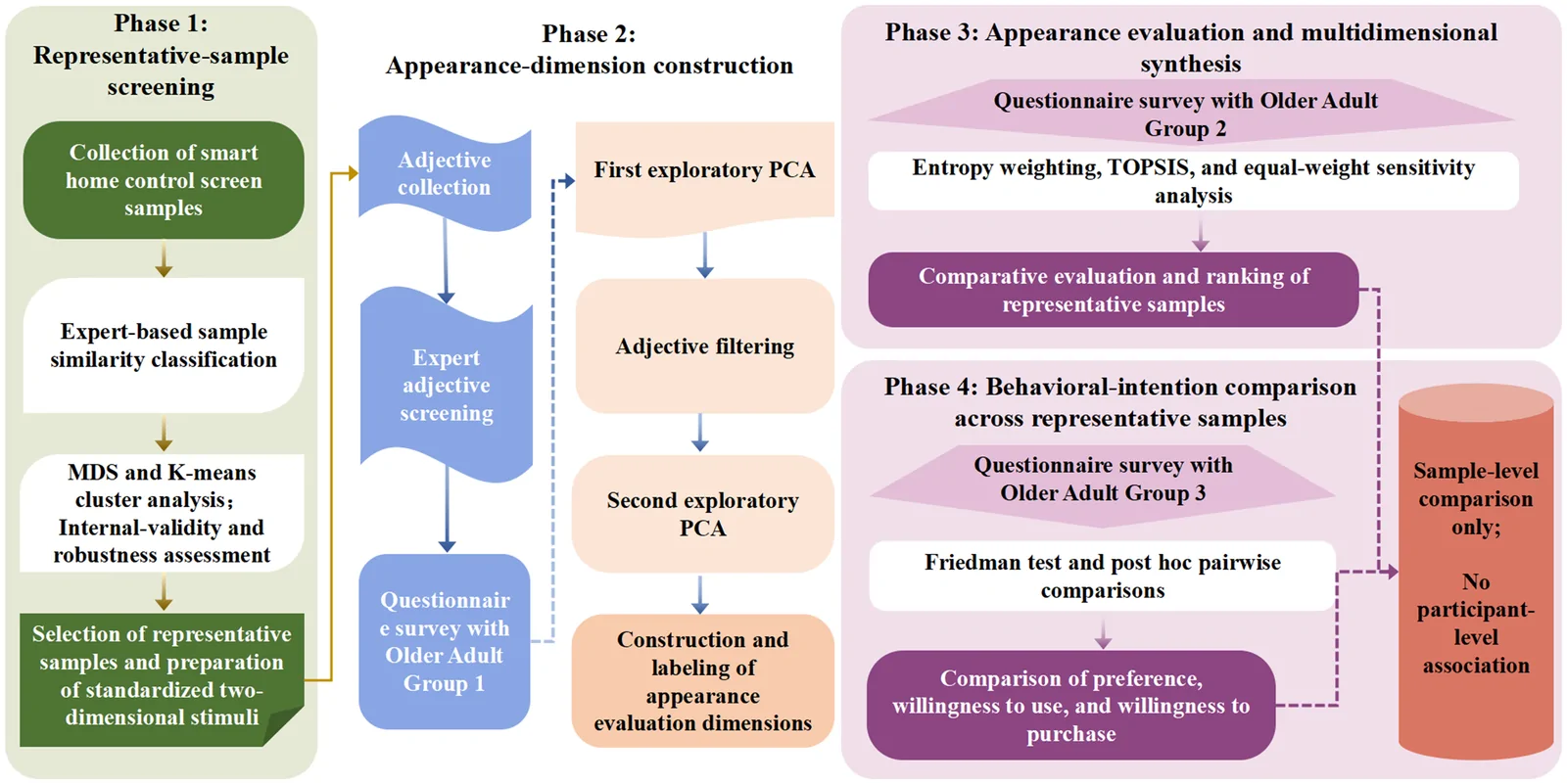
Multi-stage research framework and relationships among the four analytical phases.

To further clarify the operational sequence of the multi-stage framework, the step-by-step analytical procedure is summarized in Algorithm 1.

**Algorithm 1. Step-by-step analytical procedure of the multi-stage framework.**

Input: Smart home control screen samples, appearance-related descriptors, and stage-specific expert and older-adult evaluation data.

Output: Eight representative designs, four study-specific appearance dimensions, a composite appearance ranking, and sample-level behavioral-intention patterns.

**Step 1. Construct the initial sample pool.**

Compile smart home control-screen images from several sources, exclude duplicates and designs that cannot be distinguished visually, and preserve sufficient morphological variation for the subsequent screening.

**Step 2. Identify representative designs.**

Ask experts to assess the appearance similarity of the samples, represent the resulting dissimilarity structure with MDS, and perform K-means clustering on the retained coordinates. Evaluate the candidate solutions according to internal-validity indices, cluster-size distribution, and robustness, and retain the sample closest to the centroid of each selected cluster.

**Step 3. Standardize the evaluation stimuli.**

Prepare standardized two-dimensional versions of the selected designs by removing or simplifying color, branding, and interface content while preserving the morphological features needed for appearance evaluation.

**Step 4. Construct the appearance evaluation dimensions.**

Compile adjectives describing screen appearance, review them for relevance and semantic clarity, collect appropriateness ratings from older adults, and use exploratory PCA to derive the dimensions retained in this study.

**Step 5. Evaluate representative designs across the retained dimensions.**

Ask older adults to rate each representative design on the retained appearance dimensions, and use the mean score for each design and dimension to form the decision matrix.

**Step 6. Perform supplementary multidimensional synthesis.**

Estimate data-derived weights for the dimensions with the entropy method, incorporate them into the sample-level decision matrix, and calculate the TOPSIS closeness coefficients and ranks. Repeat the TOPSIS calculation with equal weights to examine whether the ranking depends on the weighting scheme.

**Step 7. Assess behavioral intentions.**

Ask a separate group of older adults to rate every representative design for preference, willingness to use, and willingness to purchase. Test overall differences across the repeated sample ratings with Friedman tests, followed by Nemenyi comparisons and complementary Wilcoxon signed-rank tests, with Holm adjustment conducted separately for the 28 pairwise comparisons within each outcome.

**Step 8. Compare sample-level patterns across analytical stages.**

Examine where the appearance results and behavioral-intention patterns converge or differ across the representative designs, without interpreting these comparisons as participant-level associations or causal evidence.

### Participant recruitment and ethics

Five participant groups—two expert groups and three independent groups of older adults—were prospectively recruited between 30 December 2025 and 23 January 2026. Each group participated in a specific research stage, as summarized in Table 2. Different older adults were recruited for the three evaluation stages to avoid carryover effects; consequently, data across stages were used for complementary sample-level comparisons rather than participant-level linkage. Purposive sampling supported the exploratory and task-specific design but was not intended to generate population-representative estimates. Formal measures of participants’ prior technology experience or familiarity with smart home products were not collected and therefore could not be included in the analyses. For the three older adult groups, questionnaire responses were screened before analysis. Responses were excluded if required rating items were incomplete, if the participant did not complete the full set of required sample-evaluation tasks, or if the same response option was selected for every rating item, indicating an invariant response pattern. This screening excluded 33, 16, and 23 questionnaires from Older Adult Groups 1, 2, and 3, respectively. The resulting numbers included in the analysis are reported in Table 2.

**Table 2:** Participant groups, research stages, sample sizes, included cases, and primary roles in the study.

Group	Participant type	Participants (*n*)	Included in analysis (*n*)	Research stage	Primary role / task
Expert Group 1	Experts	15	15	Stage 1	Provide similarity judgments for representative sample screening
Expert Group 2	Experts	15	15	Stage 2	Screen, group, and organize descriptive adjectives
Older Adult Group 1	Older adults	256	223		Evaluate the appropriateness of adjectives for describing appearance
Older Adult Group 2	Older adults	182	166	Stage 3	Evaluate the representative samples and provide overall ratings across the derived appearance dimensions
Older Adult Group 3	Older adults	135	112	Stage 4	Assess preference, willingness to use, and willingness to purchase

This study was reviewed and approved by the Academic Ethics and Scientific Ethics Committee of Guangdong University of Technology on 18 December 2025 (Approval No. GDUTXS20250237), before participant recruitment and data collection commenced. The Committee approved the overall study protocol, which stated that all participants would participate voluntarily after fully understanding the study purpose, procedures, and their rights, and that they could withdraw unconditionally at any stage. The approved research framework also required anonymization and data confidentiality. However, the ethics approval materials did not separately specify whether informed consent would be written or non-written and did not include a separate waiver of signed written consent. All procedures involving human participants were conducted in accordance with relevant national regulations and institutional ethical principles. The study involved only non-invasive questionnaire-based and visual-evaluation procedures and did not include physiological measurements, medical interventions, drug administration, or psychological induction.

Before participation, all participants received information concerning the study purpose, procedures, expected duration, potential risks, voluntary nature of participation, confidentiality arrangements, intended use of the data, and their right to decline participation or withdraw at any time without penalty. Participants were given an opportunity to ask questions before deciding whether to participate. When participants had difficulty reading the information, the researcher also provided an oral explanation before the evaluation began. To avoid collecting signatures as direct personal identifiers in this otherwise anonymous questionnaire-based and visual-evaluation procedure, no signed written consent forms were collected. Instead, after receiving and understanding the study information, participants indicated non-written informed consent by voluntarily choosing to proceed with and complete the questionnaire or evaluation materials. No separate personally identifiable consent record was generated, and anonymity was maintained throughout this process. This consent process was witnessed by a second member of the research team, who confirmed that every participant had received the study information, had been provided an opportunity to ask questions, and had voluntarily chosen to proceed with the corresponding questionnaire or evaluation task. No minors were included in this study. Participant anonymity and data confidentiality were maintained throughout the study, and all collected data were used solely for academic research and publication purposes.

### Screening representative samples

The sample screening process aimed to identify a manageable set of representative smart home control screen samples while reducing redundancy caused by highly similar products. The initial sample pool drew on smart home websites, e-commerce platforms, search engines, and design magazines. Direct downloads and webpage screenshots yielded 104 frontal-view images with clear visual information and little background interference. These sources were used to capture a broad range of currently available smart home control screen forms, thereby supporting the construction of a sample pool with sufficient appearance-related variation for subsequent screening. Rather than aiming for statistical representativeness of the broader market, the sample pool was constructed to maximize appearance-related variation within the present study. After duplicated or visually indistinguishable designs were removed through an internal screening process, a final dataset of 87 unique samples was retained. Each sample was sequentially coded for identification (e.g., S1 represents Sample 1).

Following the independent expert grouping task, 15 expert-specific binary dissimilarity matrices (87 × 87) were compiled and used as the direct inputs to the MDS analysis. In the archived analytical matrices, a value of 0 represented a recorded within-group similarity relationship, whereas a value of 1 represented a recorded between-group dissimilarity relationship. Because the matrices were symmetric, only the lower triangular portions were required. For presentation in S1 Data, diagonal elements were standardized to 0, while all off-diagonal binary values were retained exactly as recorded in the original SPSS file. Because the expert judgments represented categorical appearance-similarity relationships rather than direct metric distances, MDS was applied to represent the expert-derived dissimilarity structure among the samples in a reduced-dimensional space [24–26]. The archived expert-derived binary dissimilarity matrices used in the analysis are provided in S1 Data.

The analysis yielded dimensional coordinates for each sample together with model-fit indices, including Stress and R-squared (RSQ). Following the criteria proposed by Kruskal and Wish [24], Stress values of approximately 0.20, 0.10, 0.05, 0.025, and 0.00 have conventionally been characterized as indicating poor, fair, good, excellent, and perfect fit, respectively. RSQ reflects the proportion of variance in the scaled dissimilarity data accounted for by the corresponding distances in the MDS configuration, with higher values indicating better representation of the dissimilarity structure. The resulting coordinates were subjected to a series of *K*-means cluster analyses. Candidate solutions were evaluated using the average silhouette coefficient [37], Calinski–Harabasz index [38], Davies–Bouldin index [39], within-cluster sum of squares (WCSS), cluster-size distribution, preservation of appearance-related heterogeneity, and the practical number of stimuli required for the subsequent evaluation. These complementary indices were considered jointly because different internal validity measures capture different aspects of cluster compactness, separation, and partition quality [37–40]. After a cluster solution had been retained based on the joint criteria described above, its robustness was assessed through 100 repeated *K*-means analyses of the six-dimensional coordinates, using the same number of clusters as the retained solution. Run 1 used the original sample order from S1 to S87, whereas Runs 2–100 used sample permutations generated with NumPy Generator (PCG64; seed = 20260805). For each ordered dataset, the first case was selected as the first initial center, and each subsequent center was selected as the case with the greatest distance to its nearest previously selected center until *K* initial centers were obtained, where *K* corresponded to the number of clusters in the retained solution. Batch Lloyd assignment and centroid updating continued until cluster assignments no longer changed or 100 iterations were reached. Agreement between each repeated partition and the retained partition was quantified using the adjusted Rand index (ARI), which corrects the Rand index for agreement expected by chance [41,42]. The Hungarian algorithm was used to maximize membership overlap between repeated-run and retained clusters, after which cluster-retention and centroid-nearest reselection frequencies were calculated for the original representative samples. The complete run-level, sample-level, permutation-order, and cluster-matching records are provided in S1 Data.

For subsequent appearance evaluation, the frontal images of the selected representative samples were standardized and converted into two-dimensional graphics using Adobe Illustrator 2024 for Windows. As schematically illustrated in Fig 2, color information and brand identifiers were removed, interface components were simplified, and textual and iconographic elements were uniformly processed, while the original proportions and overall form characteristics were retained. This two-stage stimulus strategy served different purposes: the original product images preserved the naturally occurring visual variation required for sample screening, whereas the standardized graphics reduced the influence of color, branding, and interface content during subsequent appearance evaluation. Because clustering was not repeated after standardization, the selected designs represent the perceptual structure of the original-image sample pool rather than an independently validated standardized-stimulus space. Finally, focus group discussions and expert interviews were conducted to support the descriptive interpretation of the representative samples. Drawing on these qualitative inputs, together with prior research on morphological attributes and control modalities [14,15], several appearance-related indicators were identified to facilitate interpretation in subsequent analyses, namely size, aspect ratio, corner type, screen-to-body ratio, and button type. These indicators were introduced as descriptive tools to support later design-oriented interpretation, rather than as dimensions extracted from the MDS or clustering procedures. The selected representative samples reflect the internal structure of the present sample pool rather than the full diversity of smart home control screen designs available on the broader market.

**Fig 2:**
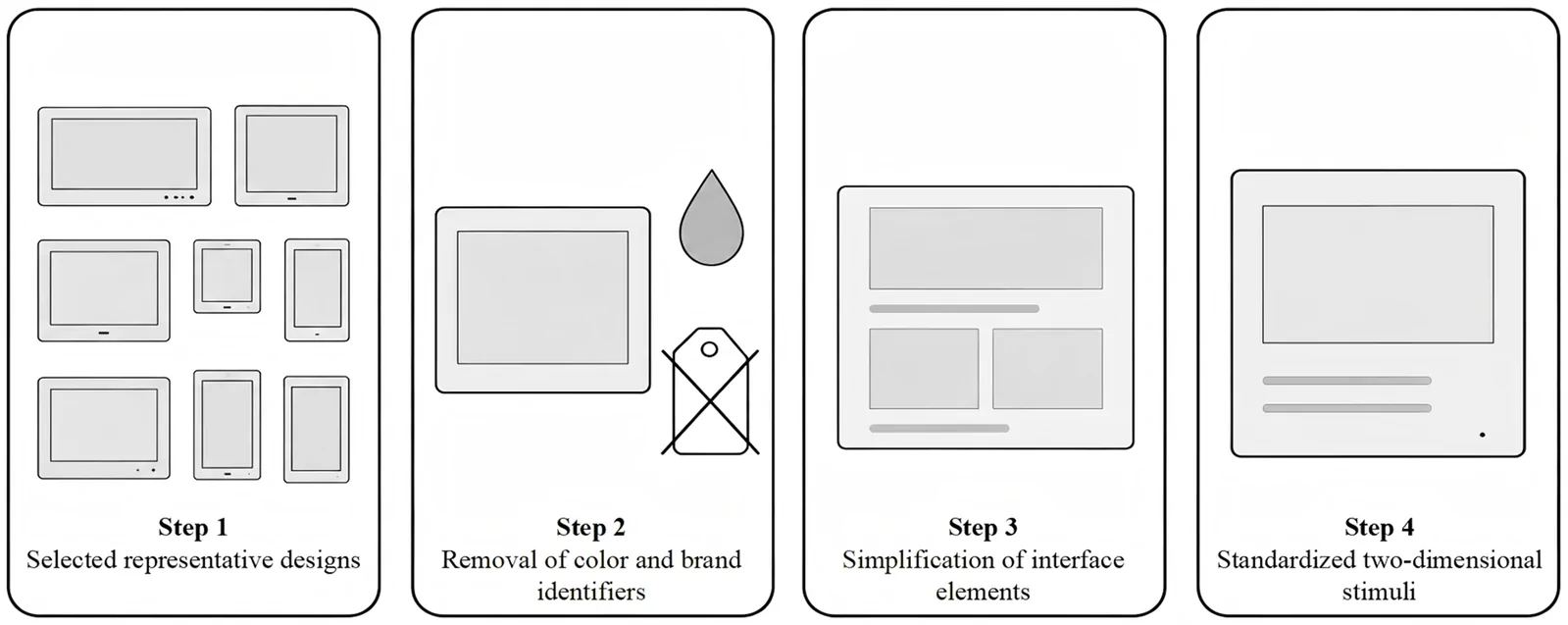
Schematic illustration of the standardization procedure used to prepare the two-dimensional evaluation stimuli.

### Construction of appearance evaluation dimensions

This stage aimed to construct appearance evaluation dimensions for smart home control screens based on representative samples. Descriptive adjectives related to the appearance of smart home control screens were first collected from multiple sources, including prior literature, product descriptions, online user comments, and expert suggestions. The research team first removed synonyms, repeated terms, and descriptors that had little relevance to appearance evaluation. A focus group then defined three selection criteria: relevance to appearance design, semantic clarity, and applicability across samples. Retained adjectives had to refer directly to appearance, be understandable to older adults, and support consistent judgments across participants. This process resulted in an initial set of 115 candidate adjectives.

Expert Group 2 evaluated whether each candidate adjective was suitable for describing the overall appearance of the representative smart home control screens. The panel included seven university faculty members in design-related fields, one senior high school Chinese-language teacher, two doctoral candidates in Chinese literature, and five doctoral candidates in design. After examining the representative samples, each expert independently identified the adjectives considered suitable. Forty adjectives selected by seven or more experts were carried forward to the subsequent evaluation.

The 40 retained adjectives were used to prepare an adjective-appropriateness questionnaire. Before data collection, participants received standardized instructions and an example explaining the evaluation task. Participants were first asked to view the representative samples and then assess the appropriateness of each adjective for describing their appearance. A representative questionnaire item was phrased as follows: “*Do you consider the adjective ‘safe’ to be appropriate for describing the appearance of these smart home control screens?*” Responses were recorded using a 5-point Likert scale ranging from 1 (*extremely inappropriate*) to 5 (*extremely appropriate*). The resulting adjective-appropriateness ratings were used in the exploratory PCA. A total of 256 questionnaires were collected from Older Adult Group 1, of which 223 were included in the analysis after data-quality screening. The analyzed sample included 136 males and 87 females; 84 participants were aged 60–65, 83 were aged 66–70, 42 were aged 71–75, and 14 were aged 76–80. The adjective appropriateness ratings used in the exploratory PCA are provided in S2 Data.

Exploratory PCA was conducted on the adjective appropriateness data to examine the grouping structure underlying older adults’ appearance evaluations. A first round of PCA was performed on the 40 retained adjectives using principal component extraction followed by Varimax rotation with Kaiser normalization. Components with eigenvalues greater than 1 were retained. Adjectives with component loadings below 0.60 were removed because they showed insufficient association with any retained component and could weaken the interpretability and internal consistency of the resulting structure. Items showing substantial cross-loadings (≥ 0.40) on more than one component were treated as problematic because they indicated ambiguous membership across components and were therefore considered for exclusion. Items loading primarily on components that were not retained in the refined structure were also excluded from subsequent analysis. After adjectives that did not meet these criteria were removed, a second round of PCA was conducted on the refined adjective set using the same extraction and rotation procedures. The resulting dimensions were named according to the component-loading patterns and semantic characteristics of the adjectives included in each grouping. Internal consistency was assessed for each dimension using Cronbach’s alpha.

### Appearance evaluation of representative samples

In this stage, older adults evaluated the representative smart home control screen samples under controlled viewing conditions. Standardized two-dimensional images of the representative samples were presented on an iPad device (248.6 mm × 179.5 mm, resolution 2360 × 1640 pixels; Fig 3A). The device size and resolution provided sufficient visual clarity for the standardized presentation of the stimuli. To minimize the influence of non-appearance-related factors on evaluation, a minimalist and neutral background was designed for sample presentation (Fig 3B), and all samples were displayed individually in randomized order. Participants were instructed to view each sample on the iPad and were allowed to zoom in and out of the images using standard touch gestures in order to examine details of form and layout according to their visual needs (Fig 3C). No time limit was imposed for viewing each sample. A total of 182 questionnaires were collected from Older Adult Group 2, of which 166 were included in the analysis after data-quality screening. The analyzed sample comprised 98 males and 68 females; 77 participants were aged 60–65, 54 were aged 66–70, 26 were aged 71–75, and nine were aged 76–80.

**Fig 3:**
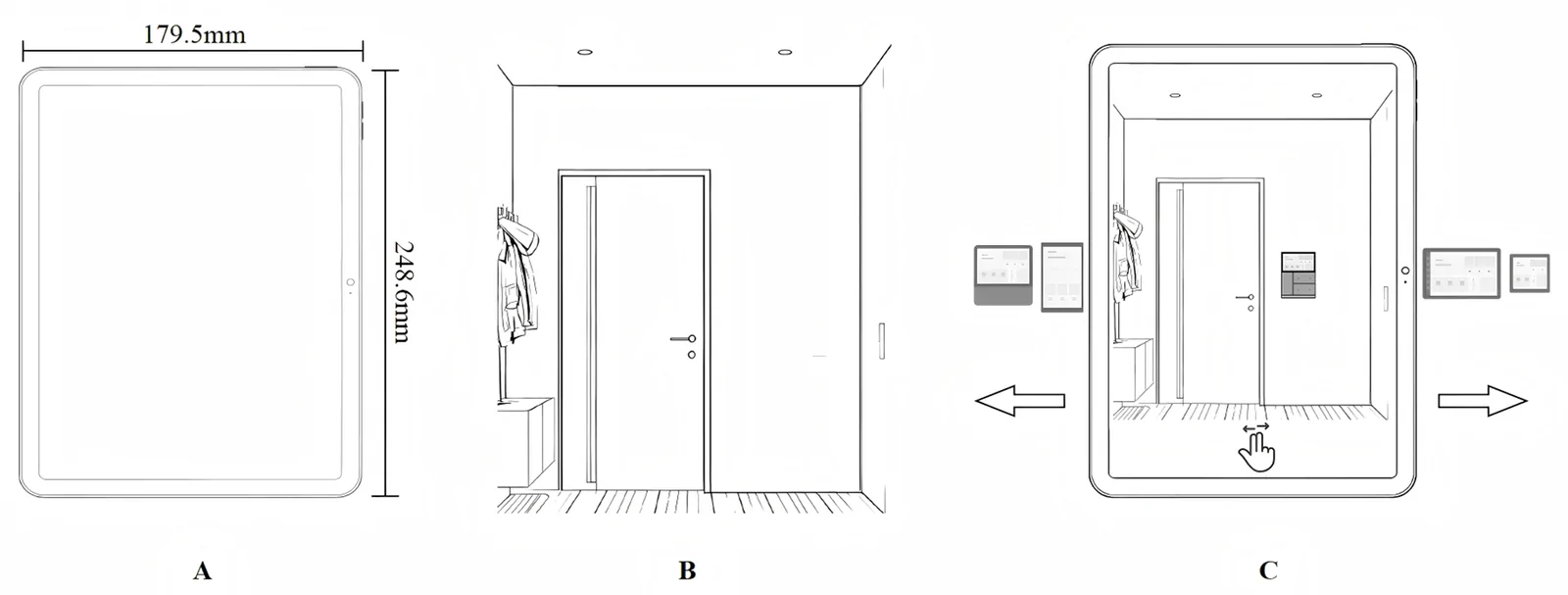
Setup for appearance evaluation. **Note.** (A) Schematic diagram of the iPad dimensions; (B) background image used for sample presentation; (C) schematic illustration of the sample-switching mechanism.

Participants completed an appearance evaluation questionnaire based on the appearance evaluation dimensions established in the previous stage. The questionnaire included ratings of each representative sample and overall ratings of the retained dimensions. The questionnaire format is illustrated by the following item: *“Sample S9: To what extent do you consider the adjectives ‘stable and reliable’ to be appropriate for describing the appearance of this smart home control screen?*” Responses were recorded using a 7-point Likert scale ranging from 1 (*extremely inappropriate*) to 7 (*extremely appropriate*). The adjective-appropriateness stage and the subsequent sample-evaluation stages served different analytical purposes and used different response scales: a 5-point scale for adjective appropriateness and a 7-point scale for ratings across representative samples. Accordingly, scores from different stages were not compared directly in absolute terms. The sample-specific ratings were used to construct the decision matrix for the TOPSIS analysis, whereas the overall dimension ratings were used to calculate the entropy-based weights. The resulting weights were then applied to the decision matrix to rank the representative designs. The sample-specific appearance evaluation ratings and overall dimension ratings used in the entropy-weighted TOPSIS analysis are provided in S3 Data. The standardized two-dimensional stimuli improved experimental control over visual-form variables by reducing extraneous variation across samples. However, this procedure was intended to isolate visual appearance judgments rather than to reproduce the multisensory and operational experience of interacting with functioning devices. Accordingly, the resulting ratings should be interpreted as controlled image-based evaluations.

### Behavioral intentions regarding representative samples

In the final stage of the study, older adults’ behavioral intentions regarding the representative smart home control screen samples were examined. Behavioral intention was treated as a comparative evaluative outcome across representative samples, and was assessed along three dimensions: preference, willingness to use, and willingness to purchase. Standardized two-dimensional images of the representative samples were presented under the same viewing conditions as those used in the previous appearance evaluation stage in order to maintain consistency across evaluation stages. A total of 135 questionnaires were collected from Older Adult Group 3, of which 112 were included in the analysis after data-quality screening. The analyzed sample included 57 males and 55 females; 40 participants were aged 60–65, 28 were aged 66–70, 19 were aged 71–75, 13 were aged 76–80, and 12 were aged 81 years or older.

After viewing each sample, participants rated the three behavioral-intention items on a 7-point Likert scale; higher scores represented stronger preference, willingness to use, and willingness to purchase. The three items were phrased as follows: “*To what extent do you like Sample S9 / are you willing to use Sample S9 / are you willing to purchase Sample S9?*” Because every participant assessed all eight representative samples, the ratings formed related repeated-measures observations across the designs. A separate Friedman test examined differences among the eight samples for each behavioral-intention outcome, and Kendall’s W was reported as the effect-size measure. When an omnibus test was significant, Nemenyi post hoc comparisons were performed. Wilcoxon signed-rank tests were additionally conducted as complementary pairwise analyses, with Holm adjustment applied separately within each behavioral intention outcome to the complete set of 28 pairwise comparisons among the eight representative samples. Statistical analyses were conducted using IBM SPSS Statistics 26.0, supplemented by the additional calculations required for the post hoc analyses. The behavioral intention ratings are provided in S4 Data.

### Use of artificial intelligence tools

During the preparation and revision of this manuscript, the authors used DeepSeek and Kimi (Moonshot AI) to assist with Chinese-to-English translation, improve language clarity and manuscript organization, and format the references. These tools were not used to generate primary research data, conduct the reported statistical analyses, or formulate the scientific interpretations and conclusions. All AI-assisted outputs incorporated into the manuscript were critically reviewed by the authors to ensure linguistic accuracy, consistency with the original content, and correct reference formatting before inclusion. The authors reviewed and approved the final manuscript and take full responsibility for its accuracy, integrity, and content.

## Results

### Screening results of representative samples

Based on the archived expert-derived binary dissimilarity matrices, MDS was applied to represent the appearance-related dissimilarity structure of the 87 smart home control screen samples in a reduced-dimensional space. As shown in Table 3, MDS solutions ranging from two to six dimensions were compared. Following the criteria proposed by Kruskal and Wish, Stress values below 0.100 indicate acceptable fit, whereas values approaching 0.050 indicate good fit. Among the tested solutions, the six-dimensional configuration yielded the lowest Stress value (0.07272) and the highest RSQ value (0.95457) and was therefore retained for subsequent cluster analysis.

**Table 3:** Stress and RSQ values of different dimensions in the results of multidimensional scaling analysis.

Dimension	2	3	4	5	6
Stress	0.12355	0.11079	0.08942	0.08193	0.07272
RSQ	0.94578	0.93874	0.94825	0.94196	0.95457

A series of *K*-means cluster analyses was conducted on the retained six-dimensional MDS coordinates. Candidate solutions were evaluated using the average silhouette coefficient, Calinski–Harabasz index, Davies–Bouldin index, WCSS, and cluster-size distribution. Across the solutions from *K* = 2 to *K* = 9, lower-order solutions showed favorable values for some internal-validity indices but produced comparatively coarse and imbalanced partitions. At *K* = 2, one cluster contained 73 of the 87 samples, while the largest cluster at *K* = 3 still contained 44 samples. As the number of clusters increased, the distribution became progressively more differentiated. A clearer improvement occurred from *K* = 7 to *K* = 8: WCSS decreased by 13.3%, from 187.701 to 162.661; the average silhouette coefficient increased from 0.244 to 0.262; the Calinski–Harabasz index increased from 23.755 to 24.939; and the Davies–Bouldin index decreased from 1.366 to 1.279. Increasing the number of clusters further to *K* = 9 reduced WCSS by only an additional 4.9%, while the corresponding internal-validity indices deteriorated. No single internal-validity index was treated as the sole decision criterion. Instead, the *K* = 8 solution was retained because it provided the clearest joint improvement from *K* = 7 across WCSS, silhouette, Calinski–Harabasz, and Davies–Bouldin measures, yielded a more differentiated cluster-size distribution than the lower-order solutions, and avoided the deterioration in internal-validity indices observed at *K* = 9, while maintaining a practical number of stimuli for subsequent evaluation. Detailed comparisons of the candidate solutions are provided in S2 Table.

The robustness of the retained solution was further examined through 100 repeated analyses using the documented case-order procedure. The adjusted Rand index had a mean of 0.682 and a median of 0.654, with a standard deviation of 0.124 and values ranging from 0.425 to 0.899. None of the repeated analyses reproduced the entire retained partition exactly, indicating moderate rather than complete partition stability. The selected representative samples remained in their corresponding matched clusters in 60%–100% of the repeated analyses, whereas exact reselection as the sample nearest to the corresponding centroid occurred in 13%–100% of the analyses. These results indicated that cluster membership was generally more stable than exact centroid-nearest selection. The retained solution produced eight clusters containing 6–18 samples. Based on the six-dimensional coordinates provided in S1 Data, distances between the final cluster centers ranged from 1.713 to 4.401. The samples nearest to the eight cluster centroids were S81, S73, S80, S9, S56, S86, S46, and S69, with centroid distances ranging from 0.629 to 1.054. The final eight-cluster solution, including cluster membership and centroid distances, is presented in Table 4.

**Table 4:** Cluster membership and centroid distances of the 87 samples.

Category /Sample (distance)							
1	2	3	4	5	6	7	8
S1 (1.320)	S5 (1.587)	S3 (1.220)	S9 (0.629)	S11 (1.168)	S6 (1.521)	S21 (1.670)	S8 (1.335)
S2 (1.013)	S7 (1.100)	S12 (1.287)	S37 (0.862)	S18 (1.200)	S16 (1.351)	S22 (1.244)	S32 (1.309)
S4 (1.214)	S10 (1.463)	S15 (1.435)	S63 (1.117)	S19 (1.405)	S28 (1.209)	S41 (1.255)	S34 (1.656)
S25 (1.171)	S13 (1.665)	S23 (1.188)	S68 (1.042)	S35 (1.639)	S33 (1.251)	S46 (0.983)	S42 (1.315)
S45 (1.202)	S14 (1.698)	S24 (1.407)	S70 (1.040)	S36 (1.619)	S40 (1.266)	S53 (1.441)	S51 (1.492)
S49 (1.254)	S17 (1.673)	S26 (1.798)	S76 (1.113)	S54 (1.448)	S43 (1.518)	S58 (1.630)	S61 (0.985)
S57 (1.953)	S20 (1.129)	S27 (1.562)		S55 (1.356)	S75 (1.659)	S78 (1.116)	S69 (0.821)
S59 (1.451)	S29 (1.552)	S38 (1.322)		S56 (1.054)	S86 (0.991)	S79 (1.763)	S87 (1.614)
S60 (1.075)	S30 (1.348)	S39 (1.551)		S84 (1.287)			
S64 (1.190)	S31 (1.639)	S52 (1.181)					
S65 (1.293)	S44 (1.612)	S66 (1.335)					
S81 (0.694)	S47 (1.500)	S71 (1.185)					
S85 (1.445)	S48 (1.258)	S72 (1.437)					
	S50 (1.431)	S74 (1.404)					
	S62 (1.918)	S77 (1.748)					
	S67 (1.576)	S80 (1.035)					
	S73 (1.000)	S82 (1.585)					
		S83 (1.295)					

**Note.** For each cluster, the sample with the shortest Euclidean distance to the corresponding centroid was selected as the representative sample.

The principal form configurations of the eight representative samples are schematically presented in Fig 4.

**Fig 4:**
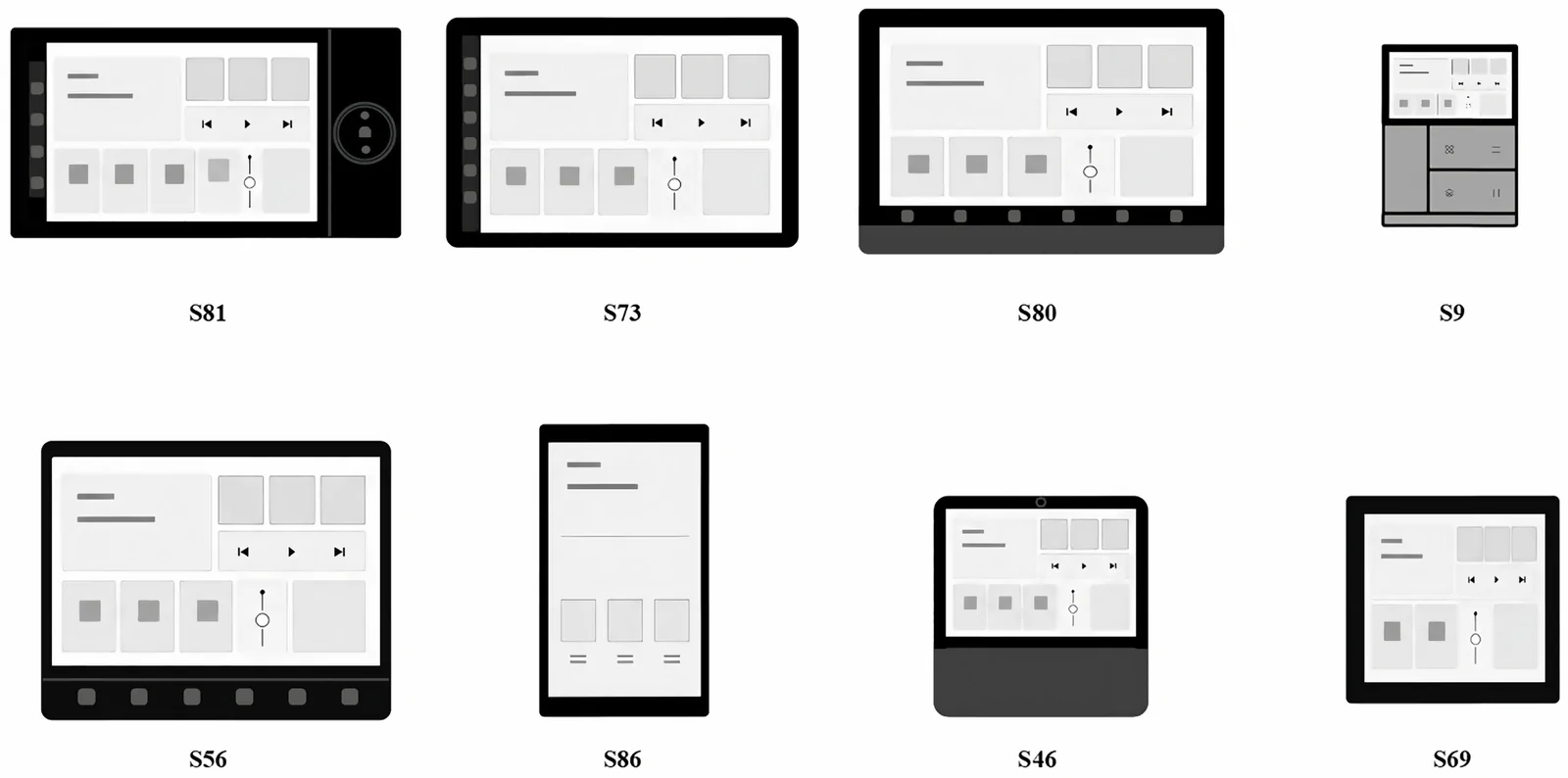
Schematic representations of the eight representative smart home control screen configurations. **Note.** The schematic representations preserve the principal morphological configurations and are not drawn to scale.

For design-level interpretation, the appearance characteristics of the representative samples were further summarized using five descriptive indicators: size, aspect ratio, corner type, screen-to-body ratio, and button type, as shown in Table 5. These indicators were introduced as descriptive tools for interpreting the representative samples rather than as dimensions extracted from the MDS solution. The eight designs therefore served as study-specific anchors reflecting the internal structure of the present sample pool rather than definitive representations of the broader market.

**Table 5:** Appearance and design features of eight representative samples.

Samples	Size	Aspect ratio	Corner type	Screen-to-body ratio	Button type
S9	Small	9:16	Small radius	Low	Physical
S46	Medium	4:3	Large radius	Medium	None
S56	Large	16:10	Medium radius	High	Physical
S69	Small	1:1	Small radius	High	None
S73	Medium	16:9	Medium radius	High	Touch
S80	Large	16:9	Medium radius	High	Touch
S81	Large	21:9	Small radius	Medium	Physical + Touch
S86	Large	9:18	Small radius	High	None

**Note.** Size was classified as small (< 5 inches), medium (5–8 inches), and large (> 8 inches). Corner radius was classified as small radius (< 2 mm), medium radius (2–4 mm), and large radius (> 4 mm). Screen-to-body ratio was categorized as low (< 65%), medium (65%–80%), and high (> 80%).

### Appearance evaluation dimensions derived from adjective appropriateness ratings

Fifteen experts independently screened the candidate adjectives for their appropriateness in describing the appearance of the eight representative smart home control screen samples. Under the retention rule, adjectives selected by at least seven experts were retained, resulting in a final set of 40 adjectives for subsequent analysis, as listed in Table 6. These adjectives were then evaluated by older adult participants in relation to the eight representative samples.

**Table 6:** Adjectives retained after expert screening for the first PCA.

Safe	Meticulous	High-quality	Durable	Practical	Clean
Technological	Balanced	Stable	Exquisite	Novel	Futuristic
Modern	Fluent	Coordinated	Readable	Proportionate	Convenient
Reliable	Tidy	Innovative	Timeless	Regular	Aesthetic
Considerate	Simple	Easy	Streamlined	Intelligent	Clear
Individualistic	Advanced	Orderly	Humanized	Efficient	Rational
Detail-oriented	High-grade	Superior	Unique		

A first round of PCA using principal component extraction and Varimax rotation with Kaiser normalization was conducted to examine the initial structure of the 40 retained adjectives. The KMO value was 0.908, and Bartlett’s test of sphericity was significant (*p* < 0.001), indicating that the data were suitable for PCA. Based on the predefined retention criteria, adjectives with low or unclear component loadings, as well as adjectives loading primarily on components that were not retained in the refined structure, were excluded after the first PCA. As a result, 22 adjectives were retained for further analysis, as shown in Table 7.

**Table 7:** Retained and excluded adjectives after the first PCA.

Adjectives retained	Component loading	Adjectives excluded	Component loading
Safe	0.804	Considerate	0.480
Technological	0.773	Balanced	−0.406
Modern	0.733	Fluent	—
Reliable	0.796	Tidy	−0.761
Meticulous	0.772	Coordinated	0.480
Simple	0.746	Durable	−0.730
High-quality	0.786	Convenient	—
Stable	0.802	Novel	−0.603
Innovative	0.734	Regular	—
Easy	0.717	Clean	−0.727
Exquisite	0.699	Streamlined	0.503
Readable	0.729	Clear	0.405
Timeless	0.708	Detail-oriented	—
Practical	0.791	High-grade	−0.701
Proportionate	0.723	Superior	—
Intelligent	0.726	Unique	—
Futuristic	0.721	Individualistic	−0.488
Aesthetic	0.737	Orderly	−0.570
Rational	0.836		
Advanced	0.713		
Humanized	0.779		
Efficient	0.843		

**Note.** The reported values represent the largest-magnitude component loadings observed in the first PCA. “—” indicates that no salient component loading was observed. Excluded adjectives either showed low or unclear loadings or loaded primarily on components that were not retained in the refined structure.

A second round of PCA was then performed on the refined adjective set using principal component extraction followed by Varimax rotation with Kaiser normalization. The KMO value increased to 0.921, and Bartlett’s test remained significant (*p* < 0.001), continuing to indicate sampling adequacy for PCA. The second PCA supported a four-component solution, accounting for 68.038% of the cumulative variance. After rotation, the retained adjectives showed clear loading patterns, with all items loading above 0.60 on a single component and no substantial cross-loadings observed (Table 8). Cronbach’s alpha values ranged from 0.887 to 0.899, indicating high internal consistency across the four dimensions (Table 9).

**Table 8:** Rotated component loadings, eigenvalues, and variance explained for the retained adjective set.

Adjective group	Adjectives	Component			
		1	2	3	4
A	Safe	0.181	0.130	0.813	0.147
	Reliable	0.133	0.106	0.811	0.187
	Stable	0.112	0.041	0.817	0.120
	Practical	0.223	0.074	0.789	0.042
	Rational	0.082	0.084	0.844	−0.002
B	Technological	0.186	0.784	0.027	0.195
	Modern	0.091	0.764	0.052	0.202
	Innovative	0.235	0.762	0.130	0.125
	Intelligent	0.174	0.764	0.091	0.101
	Futuristic	0.095	0.742	0.075	0.244
	Advanced	0.223	0.730	0.136	0.245
C	Meticulous	0.181	0.225	0.103	0.805
	High-quality	0.108	0.156	0.097	0.818
	Exquisite	0.262	0.182	0.103	0.736
	Timeless	0.200	0.259	0.094	0.714
	Aesthetic	0.222	0.245	0.125	0.772
D	Simple	0.734	0.242	0.112	0.215
	Easy	0.726	0.167	0.218	0.167
	Readable	0.747	0.188	0.165	0.208
	Proportionate	0.745	0.105	0.155	0.075
	Humanized	0.792	0.164	0.109	0.254
	Efficient	0.840	0.171	0.102	0.146
Eigenvalue		8.351	2.838	2.030	1.750
Variance explained (%)		37.958	12.898	9.229	7.952
Cumulative variance explained (%)		37.958	50.856	60.086	68.038

**Table 9:** Labels, codes, and internal consistency of the retained appearance evaluation dimensions.

Adjective group	Adjectives	Dimension Label	Code	Cronbach’s alpha
A	Safe, Reliable, Stable, Practical, Rational	*Sturdy and Reliable*	Y1	0.894
B	Technological, Modern, Innovative, Intelligent, Futuristic, Advanced	*Futuristic Technology*	Y2	0.890
C	Meticulous, High-quality, Exquisite, Timeless, Aesthetic	*High Quality*	Y3	0.887
D	Simple, Easy, Readable, Proportionate, Humanized, Efficient	*Simple and Efficient*	Y4	0.899

Based on the semantic characteristics of the retained adjective groupings, the four dimensions were labeled as *Sturdy and Reliable* (Y1), *Futuristic Technology* (Y2), *High Quality* (Y3), and *Simple and Efficient* (Y4). These dimensions formed the basis for the subsequent appearance evaluation of the representative samples.

### Supplementary synthesis of appearance evaluation results using entropy-weighted TOPSIS

Mean ratings of the eight representative designs across the four appearance evaluation dimensions were used to construct the sample-level decision matrix (Table 10). Dimension-specific results showed that S81 received the highest mean rating for *Sturdy and Reliable*, whereas S73 received the highest ratings for *Futuristic Technology*, *High Quality*, and *Simple and Efficient*. Participants’ overall ratings of the four dimensions were used separately to calculate the entropy values, degrees of diversification, and data-derived weights reported in Table 11. *Sturdy and Reliable* received the largest weight (31.3%), followed by *Simple and Efficient* (28.8%), *High Quality* (24.5%), and *Futuristic Technology* (15.4%). These weights reflect variation in the observed ratings and their relative contribution to the TOPSIS synthesis rather than subjective importance, user preference, or normative design priorities.

**Table 10:** Decision matrix for the entropy-weighted TOPSIS analysis.

Sample	Y1	Y2	Y3	Y4
S9	4.61	4.53	4.67	4.49
S46	4.77	4.77	5.02	4.55
S56	5.20	5.24	5.31	4.76
S69	4.89	4.92	4.78	4.67
S73	5.06	5.31	5.48	5.17
S80	5.13	4.96	5.17	4.90
S81	5.37	5.14	5.06	4.84
S86	4.59	4.65	4.86	5.07

**Table 11:** Entropy values, diversification degrees, and weights of the four appearance evaluation dimensions.

Criterion	Values of entropy	Degree of diversification	Weights
Y1	0.989	0.011	0.313
Y2	0.995	0.005	0.154
Y3	0.992	0.008	0.245
Y4	0.990	0.010	0.288

The derived weights were subsequently integrated with the decision matrix, and the TOPSIS procedure was applied to compute each sample’s distance to the positive and negative ideal solutions. The resulting closeness coefficients ($C_{i}^{*}$) and ranking outcomes are presented in Table 12. Within the TOPSIS framework, samples with higher $C_{i}^{*}$ values are interpreted as showing greater overall proximity to the positive ideal solution across the four appearance evaluation dimensions. In the present sample set, S73 obtained the highest$\;C_{i}^{*}$ value (0.778), indicating the highest overall composite score among the representative samples, whereas S9 showed the lowest $C_{i}^{*}$ value. These results provide a supplementary comparative view of appearance evaluation outcomes across the retained dimensions, and support subsequent discussion of sample-level similarities and differences in behavioral intention patterns.

**Table 12:** TOPSIS closeness coefficients and ranking results for the eight representative samples.

Sample	$S_{i}^{*}$	$S_{i}^{-}$	$C_{i}^{*}$	Rank
S9	0.027	0.000	0.014	8
S46	0.021	0.008	0.267	7
S56	0.010	0.020	0.668	3
S69	0.020	0.009	0.311	6
S73	0.007	0.024	0.778	1
S80	0.010	0.018	0.635	4
S81	0.010	0.021	0.675	2
S86	0.022	0.013	0.369	5

**Note.** Higher $C_{i}^{*}$ values indicate greater overall proximity to the positive ideal solution within the current sample set.

The equal-weight sensitivity analysis produced a highly similar ranking: S73, S56, S81, S80, S86, S69, S46, and S9. Compared with the entropy-weighted solution, only S56 and S81 exchanged the second and third positions, while all other ranks remained unchanged. Rank-order agreement between the two weighting schemes was high (Spearman’s ρ = 0.976), indicating that the overall ranking was relatively stable under the alternative weighting scheme.

### Behavioral intention differences across representative samples

This section reports behavioral intention differences across the eight representative smart home control screen designs shown in Fig 4. Ratings from the 112 participants included in the analysis were compared across the eight designs.

Friedman tests identified significant differences across the eight representative designs in preference (χ²(7) = 199.792, *p* < 0.001, Kendall’s *W* = 0.255), willingness to use (χ²(7) = 113.297, *p* < 0.001, Kendall’s *W* = 0.145), and willingness to purchase (χ²(7) = 168.620, *p* < 0.001, Kendall’s *W* = 0.215). The corresponding mean-rank orderings were S56 > S73 > S46 > S81 > S80 > S9 > S69 > S86 for preference; S56 > S69 > S73 > S80 > S81 > S86 > S9 > S46 for willingness to use; and S69 > S56 > S73 > S81 > S80 > S9 > S46 > S86 for willingness to purchase. Nemenyi and Holm-adjusted Wilcoxon comparisons showed broadly consistent pairwise patterns, although the Wilcoxon analyses identified additional differences between several closely ranked samples (S3 Table). These outcome-specific differences provide a basis for the subsequent sample-level comparison with appearance evaluation patterns. The Friedman test results and descriptive statistics are summarized in Tables 13 and 14, respectively.

**Table 13:** Friedman test results for behavioral intention ratings across representative samples.

Indicator	Friedman χ²	*df*	*p*	Kendall’s *W*
Preference	199.792	7	< 0.001	0.255
Willingness to Use	113.297	7	< 0.001	0.145
Willingness to Purchase	168.620	7	< 0.001	0.215

**Table 14:** Median and interquartile range (IQR) of behavioral intention ratings across representative samples.

Sample	Preference Median	Preference IQR	Willingness to Use Median	Willingness to Use IQR	Willingness to Purchase Median	Willingness to Purchase IQR
S9	6.00	2.00	5.00	1.00	4.00	4.00
S46	7.00	1.00	5.00	2.00	2.00	4.00
S56	7.00	0.00	6.00	1.00	5.50	1.00
S69	6.00	2.00	6.00	2.00	6.00	1.75
S73	7.00	0.00	6.00	1.00	5.00	2.00
S80	6.00	1.00	5.00	1.00	5.00	2.00
S81	6.00	1.00	6.00	1.00	5.00	3.00
S86	6.00	2.00	6.00	3.00	3.00	3.00

### Sample-level comparison of appearance evaluation and behavioral intention patterns

At the sample level, S73 and S56 showed relatively favorable patterns in both the entropy-weighted TOPSIS synthesis and the behavioral intention outcomes. By contrast, S81 ranked second in the TOPSIS synthesis but showed less favorable willingness-to-use and willingness-to-purchase rankings, whereas S69 ranked sixth in the TOPSIS synthesis but ranked relatively highly in willingness to use and willingness to purchase. These comparisons indicate that appearance evaluation outcomes and behavioral intention patterns did not align uniformly across the representative designs. Because the two sets of outcomes were obtained from independent participant groups, these observations are descriptive sample-level comparisons and should not be interpreted as individual-level associations.

## Discussion

### Interpretation of the multi-stage evaluation framework

This study employed a multi-stage analytical framework to examine older adults’ appearance evaluation outcomes and behavioral intention ratings for smart home control screen designs. Rather than relying on a single evaluative approach, the framework combined representative sample screening, appearance evaluation dimension construction, multidimensional synthesis, and behavioral intention comparison. Studies in other computational domains have also illustrated approaches that integrate structural relationships and semantic context when representing heterogeneous information [43,44]. In the present study, appearance dimension construction, multidimensional synthesis, and behavioral intention comparison were treated as distinct but complementary analytical stages. This organization enabled sample-level comparison across stages while maintaining a clear distinction from participant-level associations. The framework may therefore inform the organization of similar multi-stage evaluations of age-friendly interfaces.

The expanded comparison supported retaining the *K* = 8 solution as a study-specific compromise among internal validity, cluster-size distribution, preservation of appearance-related heterogeneity, and evaluation feasibility, rather than as the optimum according to any single validity index. Repeated analyses indicated moderate partition stability, with cluster membership showing greater consistency than exact centroid-nearest selection. Accordingly, the eight samples are interpreted as representing the internal appearance structure of the present sample pool rather than as universal prototypes of smart home control screens.

### Appearance evaluation dimensions and comparative contributions in the present sample set

The four appearance evaluation dimensions identified in this study—*Sturdy and Reliable* (Y1), *Futuristic Technology* (Y2), *High Quality* (Y3), and *Simple and Efficient* (Y4)—are best treated as study-specific evaluative groupings derived from the present sample and dataset, rather than as definitive or universally generalizable structures. Each dimension describes a different meaning associated with product appearance. *Sturdy and Reliable* describes visual cues of safety, stability, and dependability; *Simple and Efficient* refers to visual clarity and expected ease of use; *High Quality* captures refinement, craftsmanship, and aesthetic resolution; and *Futuristic Technology* expresses modernity, innovation, and technological symbolism. These dimensions are consistent with the organization of affective impressions into design-relevant categories in Kansei engineering [19 20] and with product-aesthetics accounts of aesthetic, semantic, and symbolic responses to visual form [21]. The prominence of reliability and comprehensibility also corresponds to age-friendly technology research emphasizing perceived ease of use and other adoption-related determinants among older adults [22,23]. Recent studies published in 2026 further indicate that older adults’ engagement with smart and digital technologies is shaped by a combination of usability, trust, facilitating conditions, emotional experience, and visual accessibility [45–47]. These literature-based parallels aid interpretation of the dimensions but do not establish them as universal constructs. Within the present framework, appearance evaluation and behavioral intention were therefore treated as complementary rather than interchangeable outcomes. Because participants’ prior technology experience and familiarity with smart home products were not measured, the present study cannot determine whether variation in these characteristics contributed to differences in appearance evaluations or behavioral intention ratings.

Entropy weighting assigned 31.3% to *Sturdy and Reliable,* 28.8% to *Simple and Efficient*, 24.5% to *High Quality*, and 15.4% to *Futuristic Technology*. In this analysis, the weights show the relative contribution of each dimension to the composite TOPSIS calculation under the observed rating structure, rather than the absolute importance that older adults assigned to each dimension. The larger contributions of Sturdy and Reliable and Simple and Efficient accord with earlier interface studies of interaction efficiency, visual search, and control performance [9,15]. The weight for *High Quality* (24.5%) was intermediate between those of *Simple and Efficient* and *Futuristic Technology* within the observed weighting structure. By contrast, the lower weight of *Futuristic Technology* indicates a smaller contribution to the entropy-weighted synthesis, but should not be interpreted as evidence that technological appearance is unimportant to older adults. The sensitivity analysis further showed that the overall ordering was largely preserved under equal weighting, with only S56 and S81 exchanging the second and third positions. The high rank-order agreement between the two weighting schemes indicates that the broad ranking pattern was relatively stable, although minor differences between closely ranked samples should be interpreted cautiously.

### Appearance characteristics and sample-level comparison of appearance evaluation and behavioral intention patterns

Comparison across the representative samples showed that certain appearance characteristics were more often observed in samples with more favorable appearance evaluation outcomes and behavioral intention patterns, although this tendency was not uniform across all samples. For example, S73 and S56 ranked relatively highly in the entropy-weighted synthesis and also showed relatively high mean ranks across the three behavioral intention outcomes. These samples shared several appearance characteristics, including relatively large display areas, high screen-to-body ratios, and comparatively balanced proportions. Previous research has shown that morphological attributes such as screen orientation and border configuration can influence visual imagery and preference [14], while control modality can affect interaction performance and visual attention [15]. In the present study, related design characteristics appeared repeatedly among the more favorably evaluated samples. At the same time, appearance evaluation outcomes and behavioral intention patterns did not always align fully across the representative samples. For instance, S81 ranked relatively high in the entropy-weighted synthesis but did not show equally high use or purchase intention, whereas S69 showed comparatively moderate appearance evaluation results but ranked higher on willingness to use and willingness to purchase. These cases indicate sample-level convergence or divergence between the two evaluation stages. However, because the outcomes were obtained from independent participant groups, they cannot be interpreted as individual-level associations or as evidence that appearance perceptions predict behavioral intentions; between-group differences may also have contributed to the observed patterns.

### Design implications for age-friendly smart home control screens

The four appearance evaluation dimensions provide a structured basis for translating the findings into design considerations for age-friendly smart home control screens. The relatively high *Sturdy and Reliable* ratings for S81, S56, S80, and S73 point to stable-looking proportions, clearly defined structural boundaries, and readily identifiable control areas. S73 and S56 received relatively high *Futuristic Technology* ratings, suggesting that screen-dominant configurations, high screen-to-body ratios, integrated displays, and restrained geometry may convey technological modernity without excessive visual complexity. The *High Quality* ratings of S73, S56, and S80 draw attention to balanced proportions and consistent treatment of bezels, corners, and other details. S73 and S86 differ in orientation and proportion but both received relatively high *Simple and Efficient* ratings, suggesting that this impression may depend more on limited visual fragmentation, a prominent main display, and clear organization of information and controls than on a single form configuration.

The contrasts among samples indicate that the dimensions should be considered together rather than treated as separate design prescriptions. Although S73 and S56 showed relatively favorable patterns across both appearance evaluation and behavioral intention, the contrasting results observed for S81, S86, and S69 suggest that no single morphological feature or appearance dimension should be treated as universally preferable. Designers may therefore consider these dimensions jointly when comparing alternative screen proportions, bezel and corner treatments, screen-to-body ratios, and control configurations. Because the present findings were derived from image-based evaluations, these design cues should be treated as screening criteria for subsequent concept development rather than as definitive real-device prescriptions. Physical or high-fidelity prototype testing with older adults would be needed to evaluate usability and operational performance.

## Conclusions, limitations and future research directions

### Conclusions

This study examined representative smart home control screen designs and older adults’ appearance evaluation outcomes and behavioral intention ratings. Using appearance similarity judgments and multidimensional scaling, eight representative designs were identified from a larger sample pool. Based on older adults’ adjective appropriateness ratings of the eight representative designs, four study-specific appearance evaluation dimensions were identified: *Sturdy and Reliable*, *Futuristic Technology*, *High Quality*, and *Simple and Efficient*. Comparative analyses further showed that the representative samples differed across both appearance evaluation outcomes and behavioral intention ratings. Some designs showed relatively favorable sample-level patterns in both stages, whereas others showed less consistent patterns across appearance evaluation and behavioral intention. Because the two stages involved different participant groups, these cross-stage similarities and differences should not be interpreted as individual-level associations between appearance evaluation and behavioral intention. Taken together, the findings offer a descriptive comparison of how the representative smart home control screen designs were evaluated within the present study context.

Methodologically, the study places representative-sample selection, empirical construction of appearance dimensions, and separate comparisons of appearance and behavioral intention within one analytical sequence. For design practice, the four study-specific dimensions offer complementary criteria for concept screening and prototype evaluation, covering visual reliability, technological expression, perceived quality, and simplicity and efficiency. In the present sample set, screen-dominant forms, relatively balanced proportions, coherent bezel and corner treatment, clearly organized controls, and limited visual fragmentation were identified as cues for further concept and prototype testing, not as generally applicable design rules.

### Limitations and future research directions

The study has several limitations. First, participants evaluated standardized two-dimensional images instead of functioning devices. This choice improved control over visual-form variables but excluded material texture, actual display characteristics, tactile feedback, physical scale, depth cues, and hands-on operating experience. Any of these omitted cues could affect perceived reliability, quality, usability, and behavioral intention during interaction with an actual device. The results should therefore be interpreted as controlled image-based evaluations and self-reported behavioral intentions rather than evidence of real-device usability, operational performance, or actual adoption behavior.

Second, original product images were used for representative-sample screening, while later evaluations used standardized graphics with color, branding, and interface details removed or simplified. Changing the stimulus format may have altered the perceptual relationships among the selected designs; clustering the standardized stimuli might therefore have produced different memberships, centroid locations, or representative selections. The later findings are consequently conditional on the representative set obtained from the original-image screening stage. Because all later evaluation stages used the same standardized stimuli under consistent viewing conditions, the uncertainty lies in transferring representativeness across stimulus formats rather than in the internal comparability of those evaluations.

Third, behavioral intention was assessed through self-reported ratings rather than observed behavior, and appearance evaluation and behavioral intention were obtained from separate participant groups. Although the use of independent groups reduced potential carryover effects, it prevented participant-level analysis of the relationship between appearance perceptions and behavioral intentions. Participants’ prior technology experience and familiarity with smart home products were not measured, and the different response scales used across stages prevent direct comparison of absolute scores. Purposive sampling may also have introduced selection bias and limits population-level generalization. In addition, the four-dimensional structure identified through exploratory PCA has not yet been independently validated through confirmatory factor analysis, and the retained clustering solution remains dependent on the original sample pool, expert-derived dissimilarity structure, MDS configuration, and clustering procedure.

Future research should compare image-based evaluations with physical or high-fidelity interactive prototypes and include task-based measures of display, tactile, and operational performance. Representative-sample screening should be repeated using standardized stimuli, or clustering solutions derived from original and standardized images should be compared directly. Using the same participants for appearance evaluation and behavioral-intention assessment would permit participant-level analysis, while field studies could test whether stated intentions correspond to actual and sustained use. Future work should measure technology experience, seek independent older-adult replication, test the prespecified four-factor model via confirmatory factor analysis, and use probability-based sampling.

## Supporting information

S1 Table Notations used in the entropy-weighted TOPSIS analysis. (DOCX)

S2 Table Internal validity and robustness assessment of the candidate *K*-means clustering solutions. (DOCX)

S3 Table Pairwise Nemenyi and Wilcoxon signed-rank comparisons of behavioral intention ratings across the eight representative samples. (DOCX)

S1 Data Archived expert-derived binary dissimilarity matrices, six-dimensional MDS coordinates, candidate *K*-means clustering solutions, and complete records for the 100-run robustness analysis of the retained *K* = 8 solution. (XLSX)

S2 Data Expert adjective-selection records and de-identified adjective appropriateness ratings used in the exploratory principal component analysis. (XLSX)

S3 Data De-identified sample-specific appearance evaluation ratings and overall dimension ratings used in the entropy-weighted TOPSIS analysis. (XLSX)

S4 Data De-identified behavioral intention ratings, including preference, willingness to use, and willingness to purchase, for the eight representative samples. (XLSX)

## References

[1] Wang PP. Population decline narrows, population quality continues to improve. National Bureau of Statistics of China. 2025. https://www.stats.gov.cn/xxgk/jd/sjjd2020/202501/t20250117_1958337.html Accessed 2026 April 28.

[2] Opinions on deepening the reform and development of elderly care services. Beijing: State Council of the People’s Republic of China. 2025. https://www.gov.cn/zhengce/202501/content_6996775.htm

[3] National Development and Reform Commission. Implementation plan for actively responding to population aging and childcare construction during the 14th five-year plan. Beijing: National Development and Reform Commission. 2024. https://www.ndrc.gov.cn/xxgk/zcfb/tz/202404/t20240403_1365464.html

[4] Ministry of Industry and Information Technology, Ministry of Civil Affairs, National Health Commission. Announcement on publishing the Directory of Smart Health and Elderly Care Products and Services Promotion (2024 Edition). Beijing: Ministry of Industry and Information Technology. 2025. https://www.hunan.gov.cn/zqt/zcsd/202506/t20250609_33702483.html

[5] Wang RJ, Lai SC, Jhuang JY, Ho MC, Shiau YC. Development of smart home gesture-based control system. Sens Mater. 2021;33:3459–71. doi: 10.18494/SAM.2021.3522

[6] Zhu J, Wang D, Zhao Y. Design of smart home environment based on wireless sensor system and artificial speech recognition. Meas Sens. 2024;33:101090. doi: 10.1016/j.measen.2024.101090

[7] Chen C-H, Lu L. Effects of input mode and screen layout on the user interface design of a smart home central control system. Displays. 2025;87:102914. doi: 10.1016/j.displa.2024.102914

[8] Yu N, Ouyang Z, Wang H, Tao D, Jing L. The Effects of Smart Home Interface Touch Button Design Features on Performance among Young and Senior Users. Int J Environ Res Public Health. 2022;19(4):2391. doi: 10.3390/ijerph19042391 35206579 PMC8872557

[9] Zhou C, Dai Y, Huang T, Zhao H, Kaner J. An empirical study on the influence of smart home interface design on the interaction performance of the elderly. Int J Environ Res Public Health. 2022;19(15):9105. doi: 10.3390/ijerph19159105 35897468 PMC9368622

[10] Zhou C, Qian Y, Huang T, Kaner J, Zhang Y. The impact of different age-friendly smart home interface styles on the interaction behavior of elderly users. Front Psychol. 2022;13:935202. doi: 10.3389/fpsyg.2022.935202 36248527 PMC9555168

[11] Krippendorff K. The semantic turn: A new foundation for design. Boca Raton (FL): CRC Press. 2005.

[12] Godovykh M. Hospitality art experience model: The effects of visual art on guests’ attitudes and behavior. Tour Hosp. 2024;5(2):439–47. doi: 10.3390/tourhosp5020027

[13] Li H, Xu J, Fang M, Tang L, Pan Y. A study and analysis of the relationship between visual-auditory logos and consumer behavior. Behav Sci (Basel). 2023;13(7):613. doi: 10.3390/bs13070613 37504059 PMC10376566

[14] Lin Z, Zhai W, Li S, Li X. Evaluating the impact of the center control touch screen of new energy vehicles on user visual imagery and preferences. Displays. 2023;78:102435. doi: 10.1016/j.displa.2023.102435

[15] Tory M, Kincaid R. Comparing physical, overlay, and touch screen parameter controls. In: Proceedings of the 2013 ACM international conference on Interactive tabletops and surfaces, St Andrews, United Kingdom. New York: Association for Computing Machinery 2013. p. 91–100. 10.1145/2512349.2512812

[16] Xue L, Yi X, Zhang Y. Research on optimized product image design integrated decision system based on Kansei engineering. Appl Sci. 2020;10(4):1198. doi: 10.3390/app10041198

[17] Zuo Y, Wang Z. Subjective product evaluation system based on Kansei engineering and analytic hierarchy process. Symmetry. 2020;12(8):1340. doi: 10.3390/sym12081340

[18] Tang CY, Fung KY, Lee EW, Ho GT, Siu KW, Mou WL. Product form design using customer perception evaluation by a combined superellipse fitting and ANN approach. Adv Eng Inform. 2013;27(3):386–94. doi: 10.1016/j.aei.2013.03.006

[19] Schütte * STW, Eklund J, Axelsson JRC, Nagamachi M. Concepts, methods and tools in Kansei engineering. Theor Issues Ergono Sci. 2004;5(3):214–31. doi: 10.1080/1463922021000049980

[20] Nagamachi M. Kansei engineering: A new ergonomic consumer-oriented technology for product development. Int J Ind Ergon. 1995;15(1):3–11. doi: 10.1016/0169-8141(94)00052-5 12164511

[21] Crilly N, Moultrie J, Clarkson PJ. Seeing things: Consumer response to the visual domain in product design. Des Stud. 2004;25(6):547–77. doi: 10.1016/j.destud.2004.03.001

[22] Pan S, Jordan-Marsh M. Internet use intention and adoption among Chinese older adults: From the expanded technology acceptance model perspective. Comput Hum Behav. 2010;26(5):1111–9. doi: 10.1016/j.chb.2010.03.015

[23] Lee C, Coughlin JF. Perspective: Older adults’ adoption of technology: An integrated approach to identifying determinants and barriers. J Prod Innov Manag. 2015;32(5):747–59. doi: 10.1111/jpim.12176

[24] Kruskal JB, Wish M. Multidimensional scaling. London: Sage Publications. 1978.

[25] Rohde DLT. Methods for binary multidimensional scaling. Neural Comput. 2002;14(5):1195–232. doi: 10.1162/089976602753633457 11972914

[26] Connor M, Lawrence AB. Understanding adolescents’ categorisation of animal species. Animals (Basel). 2017;7(9):65. doi: 10.3390/ani7090065 28867794 PMC5615296

[27] Kline P. An easy guide to factor analysis. London: Routledge. 1994.

[28] Lewis-Beck MS. Factor analysis and related techniques. Thousand Oaks (CA): Sage Publications. 1994.

[29] Shrestha N. Factor analysis as a tool for survey analysis. Am J Appl Math Stat. 2021;9(1):4–11. doi: 10.12691/ajams-9-1-2

[30] Shannon CE. A mathematical theory of communication. Bell System Technical Journal. 1948;27(4):623–56. doi: 10.1002/j.1538-7305.1948.tb00917.x

[31] Jingwen H. Combining entropy weight and TOPSIS method for information system selection. In: 2008 IEEE Conference on Cybernetics and Intelligent Systems, Chengdu, China. Piscataway (NJ): Institute of Electrical and Electronics Engineers; 2008. p. 1281–4. 10.1109/iccis.2008.4670971

[32] Oluah C, Akinlabi ET, Njoku HO. Selection of phase change material for improved performance of Trombe wall systems using the entropy weight and TOPSIS methodology. Energy and Buildings. 2020;217:109967. doi: 10.1016/j.enbuild.2020.109967

[33] Jia L-M, Tung F-W. A study on consumers’ visual image evaluation of wrist wearables. Entropy (Basel). 2021;23(9):1118. doi: 10.3390/e23091118 34573743 PMC8470360

[34] Field A. Discovering statistics using IBM SPSS statistics. 5th ed. London: SAGE Publications. 2018.

[35] Demšar J. Statistical comparisons of classifiers over multiple data sets. J Mach Learn Res. 2006;7:1–30.

[36] Holm S. A simple sequentially rejective multiple test procedure. Scand J Stat. 1979;6(2):65–70.

[37] Rousseeuw PJ. Silhouettes: A graphical aid to the interpretation and validation of cluster analysis. J Comput Appl Math. 1987;20:53–65. doi: 10.1016/0377-0427(87)90125-7

[38] Caliński T, Harabasz J. A dendrite method for cluster analysis. Commun Stat Theory Methods. 1974;3(1):1–27. doi: 10.1080/03610927408827101

[39] Davies DL, Bouldin DW. A cluster separation measure. IEEE Trans Pattern Anal Mach Intell. 1979;1(2):224–7. doi: 10.1109/tpami.1979.4766909 21868852

[40] Li J, Pan X. GPVI: An internal index based on grouping partition for clustering validity validation. Int J Comput Intell Syst. 2026;19:199. doi: 10.1007/s44196-026-01295-y

[41] Hubert L, Arabie P. Comparing partitions. J Classif. 1985;2:193–218. doi: 10.1007/BF01908075

[42] Tomo Y, Sato F, Oba M. A comparative simulation study of cluster ensemble algorithms integrated with multiple imputation for clustering with missing data. BMC Med Res Methodol. 2026;26(1):168. doi: 10.1186/s12874-026-02869-2 42226130 PMC13439842

[43] Akhtar MU, Liu J, Xie Z, Cui X, Liu X, Huang B. Multilingual entity alignment by abductive knowledge reasoning on multiple knowledge graphs. Eng Appl Artif Intell. 2025;139:109660. doi: 10.1016/j.engappai.2024.109660

[44] Akhtar MU, Liu J, Xie Z, Liu X, Ahmed S, Huang B. Entity alignment based on relational semantics augmentation for multilingual knowledge graphs. Knowledge-Based Systems. 2022;252:109494. doi: 10.1016/j.knosys.2022.109494

[45] Pirzada P, Wilde A, Doherty GH, Harris-Birtill D. Understanding older adults’ perception, acceptance, and adoption of smart home technologies. PLoS One. 2026;21(4):e0345563. doi: 10.1371/journal.pone.0345563 42013045 PMC13098919

[46] Pemberton L, Carter M, Mehandjiev N. Adoption and Use of a Smart Home Connected Care System by Older Adults: Mixed Methods Study. JMIR Aging. 2026;9:e76012. doi: 10.2196/76012 42456154 PMC13420526

[47] Ávila-Muñoz R, Alonso-González D, Arias-Astray A. Visual design and accessibility settings: An exploratory study on their role in technology adoption among older adults. Front Commun. 2026;11:1808537. doi: 10.3389/fcomm.2026.1808537

