# Supplementary material for: Older adults’ evaluations of smart home control screen designs: Appearance dimensions and behavioral intentions in a multi-stage study
Source: PLoS One. 2026 Oct 8;21(10):e0358862. doi: 10.1371/journal.pone.0358862 (PMC13649001; doi:10.1371/journal.pone.0358862)
Supplement: Supplementary file 1 [file pone.0358862.s001.docx]

**S1 Table. Notations used in the entropy-weighted TOPSIS analysis.**

| **Stage** | **Notation** | **Definition** |
| --- | --- | --- |
| General | $\text{i}$ | Index of an observational unit; a participant in the entropy-weighting step or a design alternative in the TOPSIS step |
| General | $\text{j}$ | Index of an evaluation criterion |
| General | $\text{m}$ | Number of observational units; participants in the entropy-weighting step or design alternatives in the TOPSIS step |
| General | $\text{n}$ | Number of evaluation criteria |
| Entropy weighting | $\text{X}$ | Original participant-level rating matrix used in the entropy-weighting step |
| Entropy weighting | $\text{x}_{\text{ij}}$ | Rating of participant $\text{i}$ on evaluation criterion $\text{j}$ |
| Entropy weighting | $\text{r}_{\text{ij}}$ | Normalized rating of participant $\text{i}$ on evaluation criterion $\text{j}$ |
| Entropy weighting | $\text{h}$ | Entropy normalization constant, $\text{h}$ = 1/ln (*m*) |
| Entropy weighting | $\text{e}_{\text{j}}$ | Entropy value of evaluation criterion $\text{j}$ |
| Entropy weighting | $\text{d}_{\text{j}}$ | Degree of diversification of evaluation criterion $\text{j}$, defined as $\text{d}_{\text{j}}$ = 1-$\text{e}_{\text{j}}$ |
| Entropy weighting | $\text{w}_{\text{j}}$ | Entropy-derived weight assigned to evaluation criterion $\text{j}$ |
| TOPSIS | $\text{A}_{\text{i}}$ | Design alternative $\text{A}_{\text{i}}$ |
| TOPSIS | $\text{X}_{\text{ij}}$ | Original decision-matrix value of design alternative $\text{i}$ on evaluation criterion $\text{j}$ |
| TOPSIS | $\overline{\text{X}_{\text{ij}}}$ | Normalized decision-matrix value of design alternative $\text{i}$ on evaluation criterion $\text{j}$ |
| TOPSIS | $\text{V}_{\text{ij}}$ | Weighted normalized value of design alternative $\text{i}$ on evaluation criterion $\text{j}$ |
| TOPSIS | $\text{B}$ | Set of benefit criteria |
| TOPSIS | $\text{C}$ | Set of cost criteria |
| TOPSIS | $\text{A}^{\text{*}}$ | Positive ideal solution |
| TOPSIS | $\text{A}^{\text{-}}$ | Negative ideal solution |
| TOPSIS | $\text{V}_{\text{j}}^{\text{*}}$ | Positive ideal value of evaluation criterion $\text{j}$ |
| TOPSIS | $\text{V}_{\text{j}}^{\text{-}}$ | Negative ideal value of evaluation criterion $\text{j}$ |
| TOPSIS | $\text{S}_{\text{i}}^{\text{*}}$ | Euclidean distance of design alternative $\text{i}$ from the positive ideal solution |
| TOPSIS | $\text{S}_{\text{i}}^{\text{-}}$ | Euclidean distance of design alternative $\text{i}$ from the negative ideal solution |
| TOPSIS | $\text{C}_{\text{i}}^{\text{*}}$ | Relative closeness of design alternative $\text{i}$ to the positive ideal solution |
