# Supplementary material for: Older adults’ evaluations of smart home control screen designs: Appearance dimensions and behavioral intentions in a multi-stage study
Source: PLoS One. 2026 Oct 8;21(10):e0358862. doi: 10.1371/journal.pone.0358862 (PMC13649001; doi:10.1371/journal.pone.0358862)
Supplement: Supplementary file 2 [file pone.0358862.s002.docx]

**S2 Table. Internal validity and robustness assessment of the candidate *K*-means clustering solutions.**

| **Panel A. Internal validity and structural characteristics of candidate solutions** | | | | | | | | | | | | | | | |
| --- | --- | --- | --- | --- | --- | --- | --- | --- | --- | --- | --- | --- | --- | --- | --- |
| ***K*** | **Average silhouette coefficient** | | **Calinski–Harabasz index** | | **Davies–Bouldin index** | **WCSS** | | | **Cluster sizes** | | **Cluster-size range** | | **Largest cluster (n)** | | **Largest cluster (%)** |
| 2 | 0.295 | | 26.714 | | 1.093 | 397.261 | | | 14, 73 | | 14–73 | | 73 | | 83.9% |
| 3 | 0.274 | | 30.942 | | 1.415 | 300.634 | | | 28, 15, 44 | | 15–44 | | 44 | | 50.6% |
| 4 | 0.276 | | 28.623 | | 1.301 | 256.621 | | | 41, 14, 20, 12 | | 12–41 | | 41 | | 47.1% |
| 5 | 0.261 | | 27.600 | | 1.309 | 222.523 | | | 13, 17, 12, 13, 32 | | 12–32 | | 32 | | 36.8% |
| 6 | 0.254 | | 26.933 | | 1.290 | 196.097 | | | 13, 14, 23, 19, 9, 9 | | 9–23 | | 23 | | 26.4% |
| 7 | 0.244 | | 23.755 | | 1.366 | 187.701 | | | 18, 7, 12, 13, 12, 7, 18 | | 7–18 | | 18 | | 20.7% |
| 8 | 0.262 | | 24.939 | | 1.279 | 162.661 | | | 13, 17, 18, 6, 9, 8, 8, 8 | | 6–18 | | 18 | | 20.7% |
| 9 | 0.244 | | 23.160 | | 1.455 | 154.682 | | | 13, 9, 15, 9, 7, 6, 12, 10, 6 | | 6–15 | | 15 | | 17.2% |
| **Panel B. Partition stability of the retained *K* = 8 solution across 100 repeated analyses** | | | | | | | | | | | | | | | |
| **ARI statistic** | | | | | | | | **Value** | | | | | | | |
| Mean | | | | | | | | 0.682 | | | | | | | |
| Median | | | | | | | | 0.654 | | | | | | | |
| Standard deviation | | | | | | | | 0.124 | | | | | | | |
| First quartile | | | | | | | | 0.613 | | | | | | | |
| Third quartile | | | | | | | | 0.806 | | | | | | | |
| Minimum | | | | | | | | 0.425 | | | | | | | |
| Maximum | | | | | | | | 0.899 | | | | | | | |
| Exact reproduction of the retained partition | | | | | | | | 0 | | | | | | | |
| **Panel C. Stability of the selected representative samples** | | | | | | | | | | | | | | | |
| **Sample** | | **Cluster** | | **Distance** | | | **Retention (n/100)** | | | **Retention (%)** | | **Exact reselection (n/100)** | | **Exact reselection (%)** | |
| S81 | | 1 | | 0.694 | | | 99 | | | 99% | | 96 | | 96% | |
| S73 | | 2 | | 1.000 | | | 98 | | | 98% | | 36 | | 36% | |
| S80 | | 3 | | 1.035 | | | 60 | | | 60% | | 13 | | 13% | |
| S9 | | 4 | | 0.629 | | | 100 | | | 100% | | 100 | | 100% | |
| S56 | | 5 | | 1.054 | | | 96 | | | 96% | | 56 | | 56% | |
| S86 | | 6 | | 0.991 | | | 100 | | | 100% | | 69 | | 69% | |
| S46 | | 7 | | 0.983 | | | 98 | | | 98% | | 33 | | 33% | |
| S69 | | 8 | | 0.821 | | | 86 | | | 86% | | 34 | | 34% | |

**Note.** The candidate-solution and robustness analyses were based on the six-dimensional MDS coordinates of 87 samples provided in S1 Data. Higher average silhouette and Calinski–Harabasz values and lower Davies–Bouldin values indicate more favorable internal clustering performance. WCSS naturally decreases as *K* increases and was therefore interpreted together with the other indices and cluster-size distribution. ARI = adjusted Rand index; WCSS = within-cluster sum of squares. Run 1 used the original sample order from S1 to S87, whereas Runs 2–100 used the documented sample permutations. Corresponding clusters were matched by maximum membership overlap. Cluster retention indicates the frequency with which a selected representative sample remained in its corresponding matched cluster; exact centroid-nearest reselection indicates the frequency with which it was again the sample nearest to that matched cluster centroid.
