# Supplementary material for: Older adults’ evaluations of smart home control screen designs: Appearance dimensions and behavioral intentions in a multi-stage study
Source: PLoS One. 2026 Oct 8;21(10):e0358862. doi: 10.1371/journal.pone.0358862 (PMC13649001; doi:10.1371/journal.pone.0358862)
Supplement: Supplementary file 3 [file pone.0358862.s003.docx]

**S3 Table. Pairwise Nemenyi and Wilcoxon signed-rank comparisons of behavioral intention ratings across the eight representative samples.**

| **Panel A. Preference** | | | | | |
| --- | --- | --- | --- | --- | --- |
| **Sample 1** | **Sample 2** | **Nemenyi *p*** | **Wilcoxon *Z*** | **Wilcoxon *p*** | **Holm-adjusted *p*** |
| S9 | S46 | 0.0429 | -3.030 | 0.0024 | 0.0269 |
| S9 | S56 | < 0.001 | -6.778 | < 0.001 | < 0.001 |
| S9 | S69 | 1.0000 | -2.762 | 0.0058 | 0.0403 |
| S9 | S73 | < 0.001 | -5.847 | < 0.001 | < 0.001 |
| S9 | S80 | 0.9975 | -2.953 | 0.0031 | 0.0269 |
| S9 | S81 | 0.9711 | -3.014 | 0.0026 | 0.0269 |
| S9 | S86 | 0.2982 | -0.012 | 0.9907 | 1.0000 |
| S46 | S56 | < 0.001 | -5.582 | < 0.001 | < 0.001 |
| S46 | S69 | 0.0157 | -4.109 | < 0.001 | < 0.001 |
| S46 | S73 | 0.0505 | -4.260 | < 0.001 | < 0.001 |
| S46 | S80 | 0.2410 | -2.430 | 0.0151 | 0.0760 |
| S46 | S81 | 0.4420 | -2.607 | 0.0091 | 0.0549 |
| S46 | S86 | < 0.001 | -5.049 | < 0.001 | < 0.001 |
| S56 | S69 | < 0.001 | -7.605 | < 0.001 | < 0.001 |
| S56 | S73 | 0.8960 | -3.013 | 0.0026 | 0.0269 |
| S56 | S80 | < 0.001 | -7.989 | < 0.001 | < 0.001 |
| S56 | S81 | < 0.001 | -7.191 | < 0.001 | < 0.001 |
| S56 | S86 | < 0.001 | -7.976 | < 0.001 | < 0.001 |
| S69 | S73 | < 0.001 | -7.039 | < 0.001 | < 0.001 |
| S69 | S80 | 0.9749 | -1.499 | 0.1338 | 0.4477 |
| S69 | S81 | 0.8850 | -1.590 | 0.1118 | 0.4477 |
| S69 | S86 | 0.4967 | -3.541 | < 0.001 | 0.0048 |
| S73 | S80 | < 0.001 | -6.755 | < 0.001 | < 0.001 |
| S73 | S81 | < 0.001 | -5.704 | < 0.001 | < 0.001 |
| S73 | S86 | < 0.001 | -7.505 | < 0.001 | < 0.001 |
| S80 | S81 | 1.0000 | -0.100 | 0.9201 | 1.0000 |
| S80 | S86 | 0.0591 | -4.338 | < 0.001 | < 0.001 |
| S81 | S86 | 0.0208 | -4.684 | < 0.001 | < 0.001 |
| **Panel B. Willingness to Use** | | | | | |
| **Sample 1** | **Sample 2** | **Nemenyi *p*** | **Wilcoxon *Z*** | **Wilcoxon *p*** | **Holm-adjusted *p*** |
| S9 | S46 | 0.9954 | -1.663 | 0.0962 | 0.5774 |
| S9 | S56 | < 0.001 | -7.263 | < 0.001 | < 0.001 |
| S9 | S69 | < 0.001 | -5.094 | < 0.001 | < 0.001 |
| S9 | S73 | 0.2087 | -3.736 | < 0.001 | 0.0030 |
| S9 | S80 | 0.9589 | -1.488 | 0.1367 | 0.6451 |
| S9 | S81 | 0.9710 | -0.558 | 0.5768 | 1.0000 |
| S9 | S86 | 1.0000 | -1.518 | 0.1290 | 0.6451 |
| S46 | S56 | < 0.001 | -7.008 | < 0.001 | < 0.001 |
| S46 | S69 | < 0.001 | -5.087 | < 0.001 | < 0.001 |
| S46 | S73 | 0.0280 | -4.145 | < 0.001 | < 0.001 |
| S46 | S80 | 0.5917 | -2.537 | 0.0112 | 0.1118 |
| S46 | S81 | 0.6390 | -1.920 | 0.0549 | 0.4390 |
| S46 | S86 | 0.9688 | -0.062 | 0.9506 | 1.0000 |
| S56 | S69 | 0.1310 | -3.703 | < 0.001 | 0.0032 |
| S56 | S73 | < 0.001 | -6.576 | < 0.001 | < 0.001 |
| S56 | S80 | < 0.001 | -7.135 | < 0.001 | < 0.001 |
| S56 | S81 | < 0.001 | -6.694 | < 0.001 | < 0.001 |
| S56 | S86 | < 0.001 | -6.188 | < 0.001 | < 0.001 |
| S69 | S73 | 0.5250 | -2.970 | 0.0030 | 0.0370 |
| S69 | S80 | 0.0208 | -4.551 | < 0.001 | < 0.001 |
| S69 | S81 | 0.0157 | -4.038 | < 0.001 | < 0.001 |
| S69 | S86 | < 0.001 | -4.314 | < 0.001 | < 0.001 |
| S73 | S80 | 0.8680 | -2.721 | 0.0065 | 0.0716 |
| S73 | S81 | 0.8360 | -2.984 | 0.0028 | 0.0370 |
| S73 | S86 | 0.3620 | -3.548 | < 0.001 | 0.0054 |
| S80 | S81 | 1.0000 | -0.925 | 0.3551 | 1.0000 |
| S80 | S86 | 0.9930 | -2.406 | 0.0161 | 0.1450 |
| S81 | S86 | 0.9960 | -1.815 | 0.0695 | 0.4865 |
| **Panel C. Willingness to Purchase** | | | | | |
| **Sample 1** | **Sample 2** | **Nemenyi *p*** | **Wilcoxon *Z*** | **Wilcoxon *p*** | **Holm-adjusted *p*** |
| S9 | S46 | 0.9499 | -1.924 | 0.0544 | 0.1631 |
| S9 | S56 | < 0.001 | -5.942 | < 0.001 | < 0.001 |
| S9 | S69 | < 0.001 | -7.175 | < 0.001 | < 0.001 |
| S9 | S73 | 0.0320 | -4.343 | < 0.001 | < 0.001 |
| S9 | S80 | 0.6750 | -4.322 | < 0.001 | < 0.001 |
| S9 | S81 | 0.3970 | -3.671 | < 0.001 | 0.0024 |
| S9 | S86 | 0.0930 | -3.639 | < 0.001 | 0.0025 |
| S46 | S56 | < 0.001 | -6.659 | < 0.001 | < 0.001 |
| S46 | S69 | < 0.001 | -7.648 | < 0.001 | < 0.001 |
| S46 | S73 | < 0.001 | -5.663 | < 0.001 | < 0.001 |
| S46 | S80 | 0.0830 | -4.888 | < 0.001 | < 0.001 |
| S46 | S81 | 0.0250 | -5.094 | < 0.001 | < 0.001 |
| S46 | S86 | 0.7020 | -2.799 | 0.0051 | 0.0358 |
| S56 | S69 | 0.8960 | -2.564 | 0.0104 | 0.0517 |
| S56 | S73 | 0.2550 | -3.426 | < 0.001 | 0.0050 |
| S56 | S80 | 0.0030 | -2.684 | 0.0073 | 0.0436 |
| S56 | S81 | 0.0140 | -4.701 | < 0.001 | < 0.001 |
| S56 | S86 | < 0.001 | -7.702 | < 0.001 | < 0.001 |
| S69 | S73 | 0.0060 | -5.184 | < 0.001 | < 0.001 |
| S69 | S80 | < 0.001 | -4.919 | < 0.001 | < 0.001 |
| S69 | S81 | < 0.001 | -6.356 | < 0.001 | < 0.001 |
| S69 | S86 | < 0.001 | -8.352 | < 0.001 | < 0.001 |
| S73 | S80 | 0.8290 | -0.430 | 0.6675 | 0.6675 |
| S73 | S81 | 0.9670 | -2.067 | 0.0387 | 0.1549 |
| S73 | S86 | < 0.001 | -6.563 | < 0.001 | < 0.001 |
| S80 | S81 | 1.0000 | -1.843 | 0.0653 | 0.1631 |
| S80 | S86 | < 0.001 | -6.416 | < 0.001 | < 0.001 |
| S81 | S86 | < 0.001 | -6.018 | < 0.001 | < 0.001 |

**Note.** Nemenyi tests were conducted as post hoc pairwise comparisons following significant Friedman tests. Wilcoxon signed-rank tests were conducted as complementary pairwise analyses. Within each behavioral intention outcome, Holm adjustment was applied to the complete set of 28 pairwise Wilcoxon comparisons among the eight representative samples. All reported p values are two-sided, and the Holm-adjusted values were calculated from the unrounded original p values. Values of p < 0.001 are reported as < 0.001.
