# Supplementary material for: Older adults’ evaluations of smart home control screen designs: Appearance dimensions and behavioral intentions in a multi-stage study
Source: PLoS One. 2026 Oct 8;21(10):e0358862. doi: 10.1371/journal.pone.0358862 (PMC13649001; doi:10.1371/journal.pone.0358862)
Supplement: Supplementary file 4 [file pone.0358862.s008.docx]

**PONE-D-26-21231**

**I cannot recommend this manuscript for publication in its present form.**

The manuscript does not appear to include any disclosure regarding the use of artificial intelligence, generative AI, AI-assisted writing, or AI-based language editing tools. The submission includes declarations on funding, competing interests, ethics approval, informed consent, data availability, acknowledgments, and author contributions, but no AI-use declaration is provided.

Given that the Turnitin AI-writing indicator reports 32%, the authors should be asked to provide a clear AI-use statement. This statement should specify whether any AI tools were used, the name of the tool, the purpose of use, the manuscript sections affected, and how the authors verified the accuracy, originality, references, analysis, interpretation, and conclusions. In the absence of a transparent AI-use declaration, the manuscript does not fully satisfy expected standards of disclosure and author accountability.
