# Supplementary material for: Older adults’ evaluations of smart home control screen designs: Appearance dimensions and behavioral intentions in a multi-stage study
Source: PLoS One. 2026 Oct 8;21(10):e0358862. doi: 10.1371/journal.pone.0358862 (PMC13649001; doi:10.1371/journal.pone.0358862)
Supplement: Supplementary file 5 [file pone.0358862.s009.docx]

**Response to Reviewers**

Dear Academic Editor and Reviewers,

Thank you for the careful evaluation of our manuscript and for the detailed comments. We considered each point and revised the manuscript and accompanying files where appropriate. Our responses are provided below. To make the changes easier to locate, each response identifies the relevant subsection, paragraph, and page in the revised manuscript. Page references correspond to the revised manuscript supplied with this response.

**Journal Requirements:**

**Comment 1:**

Please ensure that your manuscript meets PLOS ONE's style requirements, including those for file naming. The PLOS ONE style templates can be found at https://journals.plos.org/plosone/s/file?id=wjVg/PLOSOne_formatting_sample_main_body.pdf and <https://journals.plos.org/plosone/s/file?id=ba62/PLOSOne_formatting_sample_title_authors_affiliations.pdf>

**Response:**

Thank you for drawing our attention to these requirements. We reviewed the revised manuscript item by item against the PLOS ONE main-text and title-page templates. Specifically, we checked and standardized the title and author information, affiliations and corresponding-author details, heading hierarchy and sentence-case capitalization, double-line spacing, continuous line numbering, in-text citations of figures and tables, figure captions, table titles and notes, reference formatting, and the placement of the References and Supporting information sections. Captions for all Supporting Information files are now listed at the end of the manuscript. The manuscript, figures, tables, and Supporting Information files in the revised submission package have been assigned PLOS-compatible file names corresponding directly to their in-text labels.

Location: Throughout the revised manuscript, pp. 1–62; “Supporting information,” pp. 62; final upload package.

**Comment 2:**

In the ethics statement in the Methods, you have specified that verbal consent was obtained. Please provide additional details regarding how this consent was documented and witnessed, and state whether this was approved by the IRB.

**Response:**

Thank you for requesting further clarification of the informed-consent procedure. We have revised the wording from the potentially imprecise term “verbal consent” to “non-written informed consent” to describe the procedure implemented in this study more accurately.

Before participation, all participants received information concerning the study purpose, procedures, expected duration, potential risks, voluntary nature of participation, confidentiality arrangements, intended use of the data, and their right to decline participation or withdraw at any time without penalty. Participants were given an opportunity to ask questions before deciding whether to participate. When a participant had difficulty reading the information, the researcher also provided an oral explanation before the evaluation began. After receiving and understanding this information, participants indicated non-written informed consent by voluntarily choosing to proceed with the relevant questionnaire or visual-evaluation task. At the participant level, non-written informed consent was not documented through a signature or a separate personally identifiable consent record. Instead, it was verified through the witnessed consent procedure described below, thereby preserving the anonymity of the questionnaire-based and visual-evaluation tasks. A second member of the research team was present during the consent process and witnessed that each participant had received the study information, had been given an opportunity to ask questions, and had voluntarily chosen to proceed. We have revised the manuscript to describe this consent procedure and the witnessing arrangement explicitly.

The overall study protocol was reviewed and approved by the Academic Ethics and Scientific Ethics Committee of Guangdong University of Technology on 18 December 2025 (Approval No. GDUTXS20250237), before participant recruitment and data collection commenced. The approved protocol required informed consent, voluntary participation, the right to withdraw at any stage, anonymization, and data confidentiality. However, the ethics approval materials did not separately categorize the consent modality as written or non-written and did not include a separate waiver of signed written consent. We have therefore described the procedure that was actually implemented while avoiding any claim that exceeds the wording or scope of the ethics approval materials.

Location: “Participant recruitment and ethics,” second and third paragraphs, pp. 20–21.

**Comment 3:**

Thank you for stating the following financial disclosure: “This work was supported by the 2024 Wuxi Municipal Philosophy and Social Science Bidding Project (Social Education Development Special Fund) [grant numbers WXSK24-JY-A02]; the 2024 Jiangsu Higher Education Philosophy and Social Science Research General Project [grant numbers 2024SJYB0656].” Please state what role the funders took in the study. If the funders had no role, please state: “The funders had no role in study design, data collection and analysis, decision to publish, or preparation of the manuscript.” If this statement is not correct you must amend it as needed. Please include this amended Role of Funder statement in your cover letter; we will change the online submission form on your behalf.

**Response:**

Thank you for requesting clarification of the funders’ role. We confirm that the funders had no role in study design, data collection and analysis, the decision to publish, or preparation of the manuscript. We have therefore included the following complete statement in the revised cover letter: “This work was supported by the 2024 Wuxi Municipal Philosophy and Social Science Bidding Project (Social Education Development Special Fund) [grant number WXSK24-JY-A02] and the 2024 Jiangsu Higher Education Philosophy and Social Science Research General Project [grant number 2024SJYB0656]. The funders had no role in study design, data collection and analysis, decision to publish, or preparation of the manuscript.”

**Comment 4:**

Please provide a complete Data Availability Statement in the submission form, ensuring you include all necessary access information or a reason for why you are unable to make your data freely accessible. If your research concerns only data provided within your submission, please write “All data are in the manuscript and/or supporting information files” as your Data Availability Statement.

**Response:**

Thank you for this guidance. All de-identified data underlying the findings reported in the manuscript are now supplied directly with the submission as Supporting Information files rather than being available only on request. Specifically, S1 Data contains the dissimilarity matrices, MDS coordinates, candidate clustering results, and robustness-analysis records; S2 Data contains the adjective-selection and adjective-appropriateness records used to construct the evaluation vocabulary; S3 Data contains the appearance-rating data and overall dimension ratings used in the exploratory appearance analysis and entropy-weighted TOPSIS synthesis; and S4 Data contains the behavioral-intention ratings. We have therefore revised the manuscript and will update the submission form using the journal’s requested wording: “All data are in the manuscript and/or Supporting Information files.”

Location: “Data availability statement,” pp. 54; “Supporting information,” pp. 62.

**Comment 5:**

PLOS requires an ORCID iD for the corresponding author in Editorial Manager on papers submitted after December 6th, 2016. Please ensure that you have an ORCID iD and that it is validated in Editorial Manager. To do this, go to ‘Update my Information’ (in the upper left-hand corner of the main menu), and click on the Fetch/Validate link next to the ORCID field. This will take you to the ORCID site and allow you to create a new iD or authenticate a pre-existing iD in Editorial Manager.

**Response:**

Thank you for this reminder. The corresponding author’s ORCID iD, 0000-0002-3870-261X, has been added to Editorial Manager and authenticated through the Fetch/Validate process.

**Comment 6:**

We note that Figures 2 and 4 in your submission contain copyrighted images. All PLOS content is published under the Creative Commons Attribution License (CC BY 4.0), which means that the manuscript, images, and Supporting Information files will be freely available online, and any third party is permitted to access, download, copy, distribute, and use these materials in any way, even commercially, with proper attribution. For more information, see our copyright guidelines: <http://journals.plos.org/plosone/s/licenses-and-copyright>. We require you to either (1) present written permission from the copyright holder to publish these figures specifically under the CC BY 4.0 license, or (2) remove the figures from your submission:

1. You may seek permission from the original copyright holder of Figure(s) [#] to publish the content specifically under the CC BY 4.0 license. We recommend that you contact the original copyright holder with the Content Permission Form (http://journals.plos.org/plosone/s/file?id=7c09/content-permission-form.pdf) and the following text: “I request permission for the open-access journal PLOS ONE to publish XXX under the Creative Commons Attribution License (CCAL) CC BY 4.0 (http://creativecommons.org/licenses/by/4.0/). Please be aware that this license allows unrestricted use and distribution, even commercially, by third parties. Please reply and provide explicit written permission to publish XXX under a CC BY license and complete the attached form.” Please upload the completed Content Permission Form or other proof of granted permissions as an "Other" file with your submission. In the figure caption of the copyrighted figure, please include the following text: “Reprinted from [ref] under a CC BY license, with permission from [name of publisher], original copyright [original copyright year].”

2. If you are unable to obtain permission from the original copyright holder to publish these figures under the CC BY 4.0 license or if the copyright holder’s requirements are incompatible with the CC BY 4.0 license, please either i) remove the figure or ii) supply a replacement figure that complies with the CC BY 4.0 license. Please check copyright information on all replacement figures and update the figure caption with source information. If applicable, please specify in the figure caption text when a figure is similar but not identical to the original image and is therefore for illustrative purposes only.

**Response:**

Thank you for identifying the copyright concern. We did not retain the original product photographs or rely on third-party permission. Instead, we replaced the previous Figs 2 and 4 with new schematic figures created by the authors specifically for this study using generic geometric elements. As wholly author-created schematic figures containing no third-party material, both replacement figures are suitable for publication under the CC BY 4.0 license.

Revised Fig 2 presents the stimulus-standardization procedure without reproducing product photographs, brand identifiers, logos, or third-party interface content. Revised Fig 4 presents only the principal morphological configurations relevant to the analysis, including screen orientation, aspect ratio, bezel configuration, and physical-control arrangement. It does not reproduce original product photographs, interface screenshots, brand identifiers, logos, textual content, or any other third-party visual material. The corresponding in-text descriptions and captions now explicitly identify both figures as schematic representations.

Location: “Screening representative samples,” fourth paragraph and Fig 2 caption, pp. 24–25; “Screening results of representative samples,” paragraph following Table 4 and Fig 4 caption and note, pp. 34.

**Comment 7:**

Thank you for providing your underlying data as Supporting Information. We note that the data set contains text or data that is not in English. Please note that PLOS is an English-language publisher, so we require data sets to be provided in English as well. Please upload an English-language version of your data set. This will also allow us to determine if your data follows PLOS standards per our Data Availability policy here: <https://journals.plos.org/plosone/s/data-availability>

**Response:**

Thank you for noting this issue. We replaced the previously submitted data files with fully English-language versions and checked every worksheet for consistency. The revised files use English worksheet names, variable labels, questionnaire items, sample identifiers, response-scale descriptions, coding information, and explanatory notes.

S1 Data contains the expert-derived dissimilarity matrices, MDS coordinates, candidate clustering results, and robustness-analysis records. S2 Data contains the expert adjective-selection records and adjective-appropriateness ratings. S3 Data contains the sample-specific appearance ratings and overall dimension ratings used in the entropy-weighted TOPSIS analysis. S4 Data contains the preference, willingness-to-use, and willingness-to-purchase ratings used in the behavioral-intention analyses.

Location: Accompanying files S1 Data–S4 Data; corresponding captions in “Supporting information,” pp. 62.

**Comment 8:**

Please include captions for your Supporting Information files at the end of your manuscript, and update any in-text citations to match accordingly. Please see our Supporting Information guidelines for more information: <http://journals.plos.org/plosone/s/supporting-information.>

**Response:**

Thank you for this comment. We added a “Supporting information” section at the end of the revised manuscript containing complete captions for S1–S3 Tables and S1–S4 Data. We also checked and inserted the corresponding in-text citations at the points where each item supports a method or result. The file names, in-text labels, and end-of-manuscript captions are now consistent, allowing readers to move directly between each analysis and its associated table or dataset.

Location: S1 Table citation in “Multidimensional synthesis using entropy-weighted TOPSIS,” pp. 10; S2 Table citation in “Screening results of representative samples,” pp. 33; S3 Table citation in “Behavioral intention differences across representative samples,” pp. 43; S1 Data citations in “Screening representative samples” and “Screening results of representative samples,” pp. 22, 24, 33; S2 Data citation in “Construction of appearance evaluation dimensions,” pp. 26; S3 Data citation in “Appearance evaluation of representative samples,” pp. 29; S4 Data citation in “Behavioral intentions regarding representative samples,” pp. 31; complete captions, pp. 63.

**Comment 9:**

If the reviewer comments include a recommendation to cite specific previously published works, please review and evaluate these publications to determine whether they are relevant and should be cited. There is no requirement to cite these works unless the editor has indicated otherwise. [Note: HTML markup is below. Please do not edit.]

**Response:**

Thank you for this clarification. We read the two papers suggested by Reviewer #3 and evaluated their relevance rather than citing them automatically. Although both studies concern multilingual knowledge-graph entity alignment and do not share the present study’s application context or specific analytical procedures, they provide a useful cross-domain methodological perspective on integrating structural relationships and semantic information. We have therefore cited them in the Discussion to support the general rationale for combining complementary information across multiple analytical stages. The revised wording identifies their cross-domain relevance without implying that their application context or analytical procedures are directly equivalent to those of the present study.

Location: “Interpretation of the multi-stage evaluation framework,” first paragraph, pp. 45; References 43 and 44, p. 61.

**Reviewer #1:**

**Overall Comment :**

The manuscript addresses an important topic concerning older adults’ evaluations of smart home control screen designs and employs a multi-stage analytical framework combining multidimensional scaling, cluster analysis, exploratory factor analysis, entropy-weighted TOPSIS, and behavioral intention assessment. While the study is methodologically structured and the topic is relevant, several important issues should be addressed before the manuscript can be considered for publication.

**Response:**

We thank the reviewer for recognizing the importance of the topic and the systematic nature of the multi-stage framework. We agree that the original manuscript required stronger support for sample representativeness, clearer limits on the scope of inference, fuller theoretical interpretation, and more actionable design implications.

In response, we added a multi-index comparison of the eight-cluster solution and a 100-run robustness assessment; clarified the implications of purposive sampling, two-dimensional stimuli, and independent participant groups; expanded the theoretical interpretation of the four appearance dimensions; added an equal-weight TOPSIS sensitivity analysis; and translated the findings into testable design considerations. Our point-by-point responses below describe these changes and the corresponding interpretive limits.

**Comment 1:**

The representativeness of the selected smart home control screen samples requires stronger justification. Although MDS and cluster analysis were used to reduce the original sample pool, the rationale for selecting the final eight-cluster solution remains insufficiently supported. Additional evidence demonstrating the stability and robustness of the selected representative samples would strengthen the study.

**Response:**

Thank you for this important comment. We agree that the eight-cluster choice and the stability of the representative-sample selection required fuller support. We therefore compared candidate solutions from *K* = 2 to *K* = 9 using WCSS, average silhouette coefficient, Calinski–Harabasz index, Davies–Bouldin index, cluster-size distribution, preservation of appearance-related variation, and the practical number of later evaluation stimuli. From *K* = 7 to *K* = 8, WCSS decreased by 13.3%, the silhouette coefficient increased from 0.244 to 0.262, the Calinski–Harabasz index increased from 23.755 to 24.939, and the Davies–Bouldin index decreased from 1.366 to 1.279. Moving to *K* = 9 reduced WCSS by a further 4.9% but produced less favorable internal-validity values. We therefore describe *K* = 8 as a study-specific multi-criterion compromise rather than a universally optimal solution.

We also performed 100 repeated K-means analyses using documented sample orders. The mean ARI was 0.682 (median 0.654; range 0.425–0.899). None of the repeated analyses reproduced the complete retained partition exactly. Retention of the representative samples in their matched clusters ranged from 60% to 100%, while exact centroid-nearest reselection ranged from 13% to 100%. These results are now characterized as moderate rather than complete stability, with cluster membership generally more stable than exact centroid-nearest selection. Detailed results are reported in S2 Table. We further clarify that the eight samples represent the internal appearance structure of the present 87-image sample pool rather than the broader smart home control screen market.

Location: “Screening representative samples,” second and third paragraphs, pp. 22–23; “Screening results of representative samples,” second and third paragraphs, pp. 32–34; “Interpretation of the multi-stage evaluation framework,” second paragraph, pp. 45–46; “Limitations and future research directions,” third paragraph, pp. 53; S2 Table.

**Comment 2:**

The study relies on purposive sampling across all participant groups. The authors should discuss more explicitly how this sampling strategy may influence the generalizability of the extracted appearance evaluation dimensions and behavioral intention findings.

**Response:**

Thank you for raising this issue. We now state that purposive sampling was used to recruit participants suited to the tasks in each exploratory stage and was not intended to yield population-representative estimates. The revised limitations discussion further notes that non-probability sampling may introduce selection bias and restrict the diversity of both the expert and older-adult samples.

Accordingly, the four appearance dimensions, sample rankings, and behavioral-intention patterns are presented as exploratory findings from the current samples rather than as general characteristics of the broader older-adult population. Future work using broader, multi-site, stratified, or probability-based recruitment is proposed to examine the reproducibility of the findings across more diverse groups.

Location: “Participant recruitment and ethics,” first paragraph, pp. 18–19; “Limitations and future research directions,” third paragraph and final paragraph, pp. 53–55.

**Comment 3:**

The evaluation process was conducted using standardized two-dimensional images rather than real smart home control devices. Since appearance perception may be influenced by physical interaction, material properties, screen quality, and operational experience, the limitations associated with static visual stimuli should be discussed in greater depth.

**Response:**

Thank you for this important comment. We agree that standardized two-dimensional images cannot reproduce interaction with functioning devices. In the “Appearance evaluation of representative samples” subsection, we now explain that this format was used to control visual-form variables such as color, branding, and interface content. Accordingly, the ratings represent controlled image-based judgments rather than direct experience with real devices.

The Discussion and “Limitations and future research directions” sections now specify the omitted material texture, actual display characteristics, tactile feedback, physical scale and depth cues, and hands-on operational experience. Because these factors could affect appearance perception, usability judgments, and adoption, we have moderated statements that could be interpreted as evidence of real-device usability or actual adoption. We also recommend validation with physical or high-fidelity interactive prototypes and task-based measures such as completion time, errors, learning demands, and sustained use.

Location: “Appearance evaluation of representative samples,” first and second paragraphs, pp. 27–39; Discussion, “Design implications for age-friendly smart home control screens,” second paragraph, pp. 50; “Limitations and future research directions,” first paragraph and final paragraph, pp. 52–54.

**Comment 4:**

The four appearance evaluation dimensions derived through exploratory factor analysis are interpreted as meaningful evaluation constructs. However, stronger theoretical justification is needed to explain how these dimensions relate to existing theories and prior studies in Kansei engineering, product aesthetics, and age-friendly technology design.

**Response:**

Thank you for this valuable observation. We expanded the conceptual framework and Discussion to relate the four empirically derived dimensions to Kansei engineering, product-aesthetics accounts of aesthetic, semantic, and symbolic response, and research on older adults’ technology adoption. The revised interpretation treats *Sturdy and Reliable* as visual cues of safety, stability, and dependability; *Simple and Efficient* as visual clarity and anticipated ease of use; *High Quality* as refinement, craftsmanship, and aesthetic resolution; and *Futuristic Technology* as modernity, innovation, and technological symbolism. We also state that these theoretical correspondences assist interpretation but do not establish the four dimensions as universal constructs. Their structure should be examined further in independent samples.

Location: “Appearance evaluation and Kansei engineering,” second paragraph, pp. 6; “Appearance evaluation dimensions and comparative contributions in the present sample set,” first paragraph, pp. 46–47.

**Comment 5:**

Appearance evaluation and behavioral intention were assessed using different participant groups. Consequently, the study cannot directly examine the relationship between individual appearance perceptions and behavioral intentions. This methodological limitation should be more clearly acknowledged and discussed.

**Response:**

Thank you for emphasizing this distinction. We agree that the independent participant groups do not permit participant-level analysis of the association between appearance evaluation and behavioral intention. The research question, analytical rationale, Results, Discussion, Conclusions, and Limitations now consistently describe the cross-stage analysis as a descriptive comparison of sample-level patterns across the eight designs. The manuscript also notes that between-group differences may have contributed to convergence or divergence across stages. We therefore do not infer that an individual’s appearance perceptions predict that individual’s behavioral intentions, nor do we make participant-level causal claims. A within-participant design is proposed for future research so that individual-level associations can be examined directly.

Location: “Introduction,” Research Question 3 and the following explanatory paragraph, pp. 5–6; “Behavioral intention comparison and non-parametric analysis,” second paragraph, pp. 13; “Participant recruitment and ethics,” first paragraph, pp. 18; “Sample-level comparison of appearance evaluation and behavioral intention patterns,” pp. 44–45; “Appearance characteristics and sample-level comparison of appearance evaluation and behavioral intention patterns,” first paragraph, pp. 48–59; “Conclusions,” first paragraph, pp. 50–51; “Limitations and future research directions,” third paragraph, pp. 53.

**Comment 6:**

Although the entropy-weighted TOPSIS procedure provides a comprehensive ranking of representative samples, additional discussion is needed regarding the practical interpretation of the derived weights and whether these rankings remain stable under alternative weighting schemes.

**Response:**

Thank you for this comment. We clarified that the entropy-derived weights represent the relative variation and discriminating contribution of each dimension within the observed dataset. They should not be interpreted as the absolute importance assigned by older adults or as normative design priorities.

To examine sensitivity to the weighting scheme, TOPSIS was repeated using equal weights of 0.25 for the four dimensions. Only S56 and S81 exchanged the second and third positions, while all other ranks remained unchanged. Rank agreement was high (Spearman’s *ρ* = 0.976). We therefore describe the broad ordering as relatively stable under the equal-weight specification while noting that small differences between closely ranked designs should be interpreted cautiously.

Location: “Multidimensional synthesis using entropy-weighted TOPSIS,” second and concluding paragraphs, pp. 9–10 and 12; “Supplementary synthesis of appearance evaluation results using entropy-weighted TOPSIS,” final paragraph, pp. 42–43; “Appearance evaluation dimensions and comparative contributions in the present sample set,” second paragraph, pp. 47–48.

**Comment 7:**

The practical implications for designers and manufacturers remain relatively broad. More specific design recommendations based on the identified dimensions of “Sturdy and Reliable,” “Futuristic Technology,” “High Quality,” and “Simple and Efficient” would improve the practical value of the study.

**Response:**

Thank you for this suggestion. We added a dedicated design-implications subsection that connects each appearance dimension to specific and testable design considerations. Sturdy and Reliable is associated with stable-looking proportions, clear boundaries, and identifiable control areas. Futuristic Technology is associated with screen-dominant configurations, high screen-to-body ratios, integrated displays, and restrained geometric forms. High Quality emphasizes balanced proportions, consistent bezel and corner treatments, and refinement of visual details. Simple and Efficient emphasizes limited visual fragmentation and clear organization of information and control areas.

The contrasting results for S81, S86, and S69 are used to show that no single cue guarantees favorable appearance and behavioral-intention outcomes. The four dimensions should therefore be considered jointly and validated through real-device or high-fidelity prototype testing. The Conclusions frame these considerations as criteria for concept screening and subsequent prototype evaluation rather than as universal prescriptions.

Location: “Design implications for age-friendly smart home control screens,” pp. 49–50; “Conclusions,” second paragraph, pp. 51–52.

**Comment 8:**

Overall, the manuscript presents a potentially valuable contribution to age-friendly smart home design research. However, the issues outlined above require substantial clarification and revision.

**Response:**

We appreciate the reviewer’s overall assessment and the request for substantial clarification. In response to the seven points above, we strengthened the multi-criterion justification and robustness evidence for K = 8; clarified the limits associated with purposive sampling and static stimuli; expanded the theoretical grounding of the four appearance dimensions; restricted the cross-stage comparison to sample-level patterns; clarified the interpretation of entropy weights and added an equal-weight sensitivity analysis; and developed more specific, testable design implications.

**Reviewer #2:**

**Overall Comment :**

This is a transparent exploratory study with a solid multi-stage design (Kansei + MDS + EFA + TOPSIS).

**Response:**

We thank the reviewer for recognizing the transparency and potential value of the exploratory multi-stage design. We addressed the three observations below by distinguishing the purposes of the original photographs and standardized graphics, restricting the four dimensions and TOPSIS weights to the present samples and data, and translating the findings into design cues that can be tested in subsequent physical or high-fidelity prototypes. These changes improve the consistency between the analytical design, the scope of the conclusions, and the proposed applications.

**Comment 1:**

Sample screening used real product photos, while evaluations used simplified 2D graphics without color or branding.

**Response:**

Thank you for identifying this cross-stage difference. We now explain that the original product images preserved naturally occurring visual variation for similarity judgment and representative-sample screening, whereas standardized graphics reduced the effects of color, branding, and interface content during later evaluations.

Because the MDS configuration and clustering solution were not recalculated after standardization, the eight designs are described as representatives of the original-image sample pool rather than as independently validated representatives of the standardized-stimulus space. We also acknowledge that simplification could have altered perceptual distances, cluster membership, centroid locations, or representative selection. Future research should repeat the screening procedure using standardized stimuli or directly compare the clustering solutions obtained from the two formats.

Location: “Screening representative samples,” fourth paragraph, pp. 24–25; “Limitations and future research directions,” second paragraph and final paragraph, pp. 52–54.

**Comment 2:**

The four dimensions and TOPSIS weights are study-specific to these 8 samples, yet presented too broadly in the abstract and conclusions.

**Response:**

Thank you for this comment. We revised the Abstract and Conclusions to identify the four dimensions as specific to the present study and to frame the entropy-weighted TOPSIS results within the comparison of the eight representative designs. Phrases such as “in the present sample” and “among the eight representative designs” have been added where appropriate.

The conceptual framework and Discussion likewise avoid presenting the four dimensions as universal psychological constructs or the entropy-derived weights as general priorities. The design implications are now described as candidate cues for subsequent concept and prototype testing rather than as broadly applicable prescriptions.

Location: “Abstract,” pp. 1–2; “Appearance evaluation dimensions and exploratory principal component analysis,” second paragraph, pp. 8–9; “Appearance evaluation dimensions and comparative contributions in the present sample set,” pp. 46–48; “Conclusions,” pp. 50–52.

**Comment 3:**

The discussion of practical design implications for age-friendly interfaces remains weak.

**Response:**

Thank you for this observation. We expanded the “Design implications for age-friendly smart home control screens” subsection to connect the four dimensions to observable design variables, including screen proportions, bezel and corner treatments, screen-to-body ratios, integration between the display and device body, physical-control arrangements, and visual organization of information and control areas.

The revised discussion also uses differences among the representative samples to explain why designers should balance visual stability, technological modernity, perceived quality, and simplicity rather than optimize a single dimension in isolation. Because the present study used two-dimensional stimuli, these implications are framed as concept-screening cues that require subsequent validation using physical or high-fidelity interactive prototypes, task performance, and real-use feedback.

Location: “Design implications for age-friendly smart home control screens,” pp. 49–50; “Conclusions,” second paragraph, pp. 51–52.

**Reviewer #3:**

**Overall Comment :**

The research paper titled “Older adults’ appearance evaluation dimensions and behavioral intentions to use smart home control screens: A multi-stage exploratory analysis” discusses a research area of significant interest to the scientific community. However, the paper is currently in a suboptimal state and requires a complete overhaul before a final decision can be made. Below are few suggestions to improve the quality of the manuscript: For suggestion 1 to 7, author can refer and cite the paper titled “Multilingual entity alignment by abductive knowledge reasoning on multiple knowledge graphs” (https://doi.org/10.1016/j.engappai.2024.109660) and “Entity alignment based on relational semantics augmentation for multilingual knowledge graphs” (https://doi.org/10.1016/j.knosys.2022.109494) at a suitable location.

**Response:**

Thank you for the reviewer’s assessment of the manuscript and for the constructive suggestions. We carefully considered all seven comments and revised the relevant sections of the manuscript accordingly. Specifically, we added a brief overview of the manuscript structure at the end of the Introduction, a table defining the principal notation, an illustrative example using sample data, a comparison of the advantages and limitations of the principal analytical techniques, and a step-by-step algorithm summarizing the study procedure. We also revised the behavioral-intention analysis using statistical procedures appropriate for the repeated-measures data structure and incorporated three relevant studies published in 2026. Detailed responses to these revisions are provided point by point below.

We also reviewed the two studies recommended by the reviewer. Both studies investigate entity alignment across multilingual knowledge graphs, with particular emphasis on integrating structural relationships and semantic information to improve the representation and matching of heterogeneous entities. We therefore cited them as cross-domain methodological references to illustrate the value of integrating different types of complementary information across multiple analytical stages. These citations support the general methodological rationale for information integration and do not imply that the research objects or specific analytical procedures of the recommended studies are equivalent to those of the present study.

Location: “Interpretation of the multi-stage evaluation framework,” first paragraph, pp. 45; References 43 and 44, p. 61.

**Comment 1:**

It is suggested to briefly discuss the structure of subsequent sections in a single paragraph at the end of introduction section.

**Response:**

Thank you for this suggestion. We added a final paragraph to the Introduction that outlines the sequence and function of the remaining sections: the conceptual and analytical framework, study design and procedures, Results, Discussion, and the final Conclusions and Limitations. The paragraph also summarizes the logic of the multi-stage progression from sample screening and dimension construction to multidimensional evaluation, behavioral-intention comparison, and cross-stage interpretation.

Location: “Introduction,” final paragraph, pp. 6.

**Comment 2:**

It is suggested to include a table of notations used in the subsequent analysis.

**Response:**

Thank you for this suggestion. We added S1 Table to define the principal notation used in the entropy-weighting and TOPSIS equations, including the indices, normalized matrix, entropy values, divergence coefficients, weights, ideal solutions, distances, and closeness coefficient. An in-text citation is placed immediately before the computational steps so that readers can consult the definitions while following the formulas. Presenting the notation in a separate table improves readability without interrupting the methodological sequence.

Location: “Multidimensional synthesis using entropy-weighted TOPSIS,” second paragraph, pp. 10; S1 Table caption in “Supporting information,” pp. 62.

**Comment 3:**

It is suggested to illustrate the application of proposed study with the help of sample data in introduction section.

**Response:**

Thank you for this suggestion. We added a concise illustration using S73 in the penultimate paragraph of the Introduction. The example links the design’s visible characteristics, dimension-specific mean ratings, entropy-weighted TOPSIS closeness coefficient and rank, and the separate behavioral-intention pattern.

It therefore shows how the framework moves from design characteristics and multidimensional appearance evaluation to supplementary synthesis and sample-level cross-stage comparison. The text explicitly identifies the example as a sample-level illustration and does not imply participant-level prediction or causation.

Location: “Introduction,” penultimate paragraph, pp. 5–6.

**Comment 4:**

It is suggested to include a table in Section 2 discussing the pros and cons of popular techniques.

**Response:**

Thank you for this suggestion. We added Table 1 to compare Kansei engineering, MDS, cluster analysis, exploratory principal component analysis, entropy weighting, TOPSIS, and the Friedman/Nemenyi/Wilcoxon procedures in terms of their primary purpose, advantages, limitations, and specific role in this study.

A short paragraph before the table explains that these methods address different analytical tasks and are used complementarily rather than interchangeably. This addition makes both the rationale for the multi-stage design and the limitations of each component more transparent.

Location: “Behavioral intention comparison and non-parametric analysis,” final paragraph and Table 1, pp. 14–15.

**Comment 5:**

It is suggested to include a step-by-step algorithm outlining the proposed model.

**Response:**

Thank you for this suggestion. We added Algorithm 1 immediately after Fig 1 to summarize eight operational steps: construction of the sample pool, selection of representative designs, stimulus standardization, construction of the appearance dimensions, appearance evaluation, supplementary multidimensional synthesis, behavioral-intention assessment, and sample-level comparison across stages.

The algorithm identifies the principal input, operation, and output of each step, and the final step explicitly excludes participant-level association and causal inference. It therefore provides a concise roadmap that corresponds to the Methods, data files, and Results sections.

Location: “Study design and procedures,” immediately after Fig 1 (Algorithm 1), pp. 16–18.

**Comment 6:**

It is suggested to perform statistical test such as Friedman test, Nemenyi test and Wilcoxon test.

**Response:**

Thank you for this methodological suggestion. We re-examined the behavioral-intention data structure and confirmed that each participant rated all eight designs. The observations therefore constituted related repeated measures rather than independent-group data. We replaced the independent-group procedure with separate Friedman tests for preference, willingness to use, and willingness to purchase. Significant omnibus tests were followed by Nemenyi comparisons. Wilcoxon signed-rank tests with Holm adjustment across the 28 pairwise comparisons within each outcome were additionally reported as complementary analyses. Kendall’s W was reported as the effect-size measure.

The revised analyses showed significant differences among the eight designs for preference, willingness to use, and willingness to purchase: χ²(7) = 199.792, 113.297, and 168.620, respectively; all *p* < 0.001. Kendall’s W values were 0.255, 0.145, and 0.215. Detailed pairwise results are supplied in S3 Table. We also revised the methodological rationale, Table 1, Algorithm 1, the behavioral-intention Methods and Results, and the corresponding Discussion so that the statistical procedures are consistent with the repeated-measures design.

Location: “Behavioral intention comparison and non-parametric analysis,” pp. 12–15; “Behavioral intentions regarding representative samples,” second paragraph, pp. 30–31; “Behavioral intention differences across representative samples,” pp. 43–44; S3 Table.

**Comment 7:**

It is suggested to include at least 3 new papers from 2026.

**Response:**

Thank you for suggesting that we consider the most recent literature. We added three studies published in 2026 that are directly relevant to older adults’ adoption of smart-home or digital technologies, smart-home connected-care systems, and the role of visual accessibility in technology adoption.

These studies update the Discussion of usability, facilitating conditions, visual accessibility, appearance evaluation, and behavioral intention. They are used to contextualize, rather than determine, the interpretation of our results. The corresponding references have been added to the reference list.

Location: “Appearance evaluation dimensions and comparative contributions in the present sample set,” first paragraph, pp. 47; References 45–47, pp. 61.

**Reviewer #4:**

**Overall Comment :**

This is a well-written and relevant study addressing an important topic in age-friendly smart home design. The multi-stage research design is systematic, and the statistical analyses are generally appropriate. The manuscript clearly presents the research questions, methods, results, and limitations.

**Response:**

Thank you for the reviewer’s positive assessment of the manuscript and for recognizing the relevance of the research topic, the systematic multi-stage study design, and the clarity of the presentation. We carefully considered the major and minor comments below and revised the manuscript to strengthen data accessibility, ecological-validity boundaries, the interpretation of cross-stage stimulus differences, the scope of inference from independent participant groups, the justification of the clustering solution, and the directions for subsequent validation.

**Major comment 1:**

The Data Availability Statement should be strengthened. The manuscript states that data are available upon reasonable request, which may not fully meet PLOS ONE's data-sharing requirements. Consider depositing the dataset in a public repository and providing a DOI or link.

**Response:**

Thank you for this suggestion. The data underlying the reported analyses are no longer described as available only upon request. All de-identified data required to reproduce the principal analyses are now supplied directly with the submission as S1–S4 Data. Specifically, S1 Data contains the dissimilarity matrices, MDS coordinates, candidate clustering results, and robustness-analysis records; S2 Data contains the adjective-selection and adjective-appropriateness records used to construct the evaluation vocabulary; S3 Data contains the appearance-rating data and overall dimension ratings used in the exploratory appearance analysis and entropy-weighted TOPSIS synthesis; and S4 Data contains the behavioral-intention ratings.

Because the complete underlying datasets are provided as downloadable and extractable Supporting Information files, we did not deposit a duplicate copy in a separate repository and do not claim that a DOI is available. The Data Availability Statement has been revised to identify the manuscript and Supporting Information as the actual access route: “All data are in the manuscript and/or Supporting Information files.”

Location: “Data availability statement,” pp. 54–55; “Supporting information,” pp. 62; accompanying S1 Data–S4 Data files.

**Major comment 2:**

The study relies on standardized 2D images rather than real interactive devices. While this improves experimental control, it limits ecological validity and should be emphasized further.

**Response:**

Thank you for this comment. We now state more directly that standardized two-dimensional images improve control over visual-form variables but cannot reproduce the multisensory and operational experience of real devices. The revised text identifies the missing material, display, tactile, scale, depth, and interaction cues and limits the interpretation to controlled image-based judgments and self-reported intentions.

Physical or high-fidelity prototype testing and task-based measures are proposed as the next validation steps. The manuscript no longer treats the present findings as direct evidence of real-device usability, actual adoption, or sustained use.

Location: “Appearance evaluation of representative samples,” first and second paragraphs, pp. 27–29; “Design implications for age-friendly smart home control screens,” second paragraph, pp. 50; “Limitations and future research directions,” first paragraph and final paragraph, pp. 52–54.

**Major comment 3:**

Representative samples were selected from original product images, whereas evaluations used simplified renderings. The potential impact of this difference on the results should be discussed more thoroughly.

**Response:**

Thank you for drawing attention to this issue. We clarified the different purposes of the original product photographs and standardized graphics. The photographs preserved naturally occurring visual variation for similarity judgment and representative-sample screening, whereas the standardized graphics reduced the influence of color, branding, and interface content during subsequent evaluations.

Because MDS, clustering, and centroid-nearest selection were not repeated after standardization, we acknowledge that simplification could have altered perceptual distances, cluster membership, centroid locations, or representative selection. The eight designs are therefore interpreted as representatives of the original-image sample pool.

Because all subsequent appearance and behavioral-intention evaluations used the same standardized stimuli, the principal uncertainty concerns the transfer of representativeness between stimulus formats rather than comparability within the later stages. We added a future-research recommendation to repeat the screening procedure using standardized stimuli or directly compare the clustering results obtained from both formats.

Location: “Screening representative samples,” fourth paragraph, pp. 24–25; “Limitations and future research directions,” second paragraph and final paragraph, pp. 52–54.

**Major comment 4:**

Appearance evaluation and behavioral intention were collected from different participant groups. Therefore, relationships between these constructs cannot be directly inferred and should be interpreted cautiously.

**Response:**

Thank you for emphasizing this point. The manuscript now consistently states that appearance evaluation and behavioral intention were obtained from independent participant groups and may be compared only as sample-level patterns across the eight designs.

We do not infer that an individual’s appearance perceptions predict that individual’s behavioral intentions, and the Discussion notes that between-group differences may have influenced the observed convergence or divergence. Future research using joint or within-participant measurement is proposed to permit participant-level association analysis.

Location: “Introduction,” Research Question 3 and following paragraph, pp. 5–6; “Behavioral intention comparison and non-parametric analysis,” second paragraph, pp. 13; “Participant recruitment and ethics,” first paragraph, pp. 18–19; “Sample-level comparison of appearance evaluation and behavioral intention patterns,” pp. 44–45; “Appearance characteristics and sample-level comparison of appearance evaluation and behavioral intention patterns,” pp. 48–49; “Conclusions,” first paragraph, pp. 50–51; “Limitations and future research directions,” third paragraph and final paragraph, pp. 53–54.

**Minor comment 1:**

Provide stronger justification for the selection of the eight-cluster solution.

**Response:**

Thank you for this suggestion. We strengthened the justification for *K* = 8 by combining internal-validity indices with cluster-size distribution, preservation of appearance-related heterogeneity, later evaluation feasibility, and a documented 100-run stability assessment.

Across *K* = 2–9, the transition from *K* = 7 to *K* = 8 reduced WCSS by 13.3% and improved the silhouette, Calinski–Harabasz, and Davies–Bouldin indices. By contrast, *K* = 9 reduced WCSS by only a further 4.9% and produced less favorable internal-validity values. In the repeated analyses, the mean ARI was 0.682, representative-sample cluster retention ranged from 60% to 100%, and exact centroid-nearest reselection ranged from 13% to 100%.

Location: “Screening representative samples,” pp. 21–25; “Screening results of representative samples,” pp. 31–32; “Interpretation of the multi-stage evaluation framework,” second paragraph, pp. 45–46; S2 Table.

**Minor comment 2:**

Consider reporting participants’ technology experience or familiarity with smart home products, as these factors may influence evaluations.

**Response:**

Thank you for this helpful suggestion. We agree that participants’ prior technology experience and familiarity with smart-home products may influence their appearance evaluations and behavioral-intention ratings. Because the data collection in the present study focused primarily on appearance evaluation and behavioral intention, prior technology experience and familiarity with smart-home products were not included among the measured participant characteristics. Therefore, the potential influence of these factors could not be examined in the present analyses.

We have clarified the scope of the participant information collected in the “Participant recruitment and ethics” subsection. We have also added a corresponding discussion noting that differences in prior technology experience or familiarity with smart-home products may have contributed to variation in the evaluation results. Future studies should incorporate explicit measures of these characteristics and further examine their associations with appearance evaluation and behavioral intention.

Location: “Participant recruitment and ethics,” first paragraph, pp. 18–19; “Appearance evaluation dimensions and comparative contributions in the present sample set,” first paragraph, pp. 46–47; “Limitations and future research directions,” third paragraph and final paragraph, pp. 53–54.

**Minor comment 3:**

Future studies could validate the four-factor structure using confirmatory factor analysis and investigate real-world usage behavior.

**Response:**

Thank you for this suggestion. We now clarify that the four-component solution was obtained through exploratory principal component analysis in the present sample and has not been independently replicated. Future research should first examine whether the same dimensional pattern can be reproduced in an independent older-adult sample and then evaluate an explicitly specified four-factor measurement model using confirmatory factor analysis, including factor loadings, model fit, reliability, and measurement stability.

We also recommend field- or task-based studies using functioning devices or high-fidelity prototypes to examine whether self-reported intentions correspond to observed task performance, sustained use, and longer-term adoption. These revisions distinguish structural validation from external behavioral validation.

Location: “Limitations and future research directions,” third paragraph and final paragraph, pp. 53–54.

**Concluding comment:**

Overall, the manuscript makes a useful contribution and could be suitable for publication after addressing the above concerns.

**Response:**

Thank you for the reviewer’s positive overall assessment of the manuscript and for recognizing its potential contribution to age-friendly smart home design research. We appreciate the constructive comments and suggestions provided throughout the review. We have carefully addressed the concerns raised above and revised the relevant sections of the manuscript accordingly, including the clarification of data availability, ecological validity, cross-stage stimulus differences, interpretation of findings obtained from separate participant groups, justification of the eight-cluster solution, consideration of participants’ technology experience and smart home familiarity, and directions for confirmatory factor analysis and real-world behavioral validation. We are grateful for these suggestions, which have helped us further refine the manuscript.

**Reviewer #5:**

**Overall Comment :**

The manuscript addresses a relevant topic related to older adults’ evaluation of smart home control screen appearance and behavioral intention. The multi-stage design, including sample screening, multidimensional scaling, exploratory factor analysis, entropy-weighted TOPSIS, and non-parametric testing, is potentially useful for age-friendly interface design research.

**Response:**

Thank you for recognizing the relevance of the research topic and the potential value of the multi-stage analytical framework. We appreciate the reviewer’s positive assessment of the study’s combination of representative-sample screening, multidimensional scaling, exploratory principal component analysis, entropy-weighted TOPSIS, and non-parametric testing. In the revised manuscript, we have further clarified the distinct role of each analytical stage and how these stages collectively support the comparison of older adults’ appearance evaluations and behavioral-intention patterns across the representative designs. We have also refined the discussion of the study’s implications for age-friendly interface concept screening and subsequent prototype evaluation.

**Comment 1:**

However, I cannot recommend acceptance of the manuscript in its present form because of a significant transparency and publication-ethics concern. The Turnitin AI-writing indicator reports 32% AI-generated content, but the manuscript does not provide any clear declaration regarding the use or non-use of artificial intelligence, generative AI, AI-assisted writing, AIbased language editing, or machine translation tools. The submission includes statements on funding, competing interests, ethics approval, informed consent, data availability, acknowledgments, and author contributions, but no AI-use statement is provided.

**Response:**

Thank you for raising this important concern regarding research transparency and publication ethics. We agree that the use of artificial intelligence tools during manuscript preparation should be explicitly disclosed. We acknowledge that the previous version did not include a sufficiently clear AI-use statement and sincerely apologize for this omission. To address this concern, we have added a dedicated statement under the heading “Use of artificial intelligence tools” at the end of the Methods section, immediately before the Results section, in the revised manuscript.

During the preparation and revision of this manuscript, the authors used DeepSeek and Kimi (Moonshot AI) to assist with Chinese-to-English translation, improve language clarity and manuscript organization, and format the references. These tools were not used to generate primary research data, conduct the reported statistical analyses, or formulate the scientific interpretations and conclusions. All AI-assisted outputs incorporated into the manuscript were critically reviewed by the authors to ensure linguistic accuracy, consistency with the original content, and correct reference formatting before inclusion. The authors reviewed and approved the final manuscript and take full responsibility for its accuracy, integrity, and content.

Location: “Use of artificial intelligence tools,” pp. 31.
