# Supplementary material for: Older adults’ evaluations of smart home control screen designs: Appearance dimensions and behavioral intentions in a multi-stage study
Source: PLoS One. 2026 Oct 8;21(10):e0358862. doi: 10.1371/journal.pone.0358862 (PMC13649001; doi:10.1371/journal.pone.0358862)
Supplement: Supplementary file 6 [file pone.0358862.s010.docx]

**Response to Reviewers**

Dear Editors and Reviewers,

We sincerely thank the editorial team and all reviewers for their careful evaluation of our manuscript and for the constructive comments and suggestions provided during the review process. We have carefully considered the comments received in this round and have made corresponding revisions to the manuscript and accompanying materials. The details of our responses are provided below.

**Response to the Academic Editor****:**

**Comment 1:**

Thank you for submitting your manuscript to PLOS One. After careful consideration, we feel that it has merit but does not fully meet PLOS One’s publication criteria as it currently stands. Therefore, we invite you to submit a revised version of the manuscript that addresses the points raised during the review process.

**Response:**

Thank you for the opportunity to revise our manuscript. We have carefully addressed the Academic Editor’s comments and have prepared a revised manuscript, a marked-up version with changes highlighted, and this response letter for resubmission.

**Comment 2:**

Kindly consider making the results, codebase, and reproducibility package publicly available online. Please ensure that the repository contains all materials required to reproduce the reported findings.

**Response:**

Thank you for this helpful suggestion. We have deposited a reproducibility package in Zenodo and updated the Data Availability Statement accordingly. The package includes the de-identified datasets, supporting tables, analysis scripts, questionnaire and stimulus-material documentation, figure files, individual standardized stimulus images, and a reproducibility workflow. The repository is available at: https://doi.org/10.5281/zenodo.22303716

**Comment 3:**

In addition, verify that all equations are clearly presented, consistently formatted, and properly cited when adopted from prior work. For example, Equation (1) is not presented clearly and should be revised to improve readability and attribution, where applicable.

**Response:**

Thank you for this comment. We have checked all equations in the manuscript for clarity, consistency of formatting, and source attribution where applicable. Equation (1) has been reformatted to improve readability, and the methodological source of the procedure associated with Equation (1) has been properly cited in the accompanying text. Equations adopted from established analytical procedures have also been linked to the relevant methodological citations where applicable.

**Comment 4:**

If you would like to make changes to your financial disclosure, please include your updated statement in your cover letter.

**Response:**

Thank you for this reminder. We have carefully reviewed the financial disclosure statement and confirm that no changes are needed at this stage.

**Comment 5:**

Guidelines for resubmitting your figure files are available below the reviewer comments at the end of this letter.

**Response:**

Thank you for this reminder. We have reviewed the journal’s figure-file guidelines and prepared the figure files for resubmission accordingly. The figures have been named consistently with the in-text citations and captions and will be uploaded separately through the submission system as required.

**Comment 6:**

If applicable, we recommend that you deposit your laboratory protocols in protocols.io to enhance the reproducibility of your results.

**Response:**

Thank you for this helpful recommendation. We have carefully considered its applicability to the present study. Since the study was based on questionnaire-based evaluations of standardized visual stimuli and did not involve laboratory protocols, biological experiments, clinical procedures, or laboratory-based interventions, we believe that deposition in protocols.io is not applicable in this case.

**Comment 7:**

As the corresponding author, your ORCID iD is verified in the submission system and will appear in the published article. PLOS supports the use of ORCID, and we encourage all coauthors to register for an ORCID iD and use it as well.

**Response:**

Thank you for this reminder. We are pleased to confirm that the corresponding author’s ORCID iD has been verified in the submission system. We will also remind all coauthors to verify their ORCID iDs in the submission system, where applicable.

**Journal Requirements:**

**Comment 1:**

If the reviewer comments include a recommendation to cite specific previously published works, please review and evaluate these publications to determine whether they are relevant and should be cited. There is no requirement to cite these works unless the editor has indicated otherwise.

**Response:**

Thank you for this clarification. We have reviewed and evaluated the publications previously suggested by the reviewer before deciding whether they should be cited. As these studies provide a useful cross-domain methodological perspective, we retained the relevant citations in the Discussion to support the broader rationale for integrating complementary information across multiple analytical stages.

**Comment 2:**

Please review your reference list to ensure that it is complete and correct. If you have cited papers that have been retracted, please include the rationale for doing so in the manuscript text, or remove these references and replace them with relevant current references. Any changes to the reference list should be mentioned in the rebuttal letter that accompanies your revised manuscript. If you need to cite a retracted article, indicate the article’s retracted status in the References list and also include a citation and full reference for the retraction notice.

**Response:**

Thank you for this reminder. We have carefully reviewed the reference list to ensure that it is complete and accurate. During this check, we identified and corrected an issue in Reference 4, where the source attribution and bibliographic details of the 2024 Edition of the Directory of Smart Health and Elderly Care Products and Services Promotion required clarification. The reference has been revised to identify the joint issuing bodies and the final announcement more accurately. We also checked the cited publications for possible retraction notices using DOI- and title-based searches, and no retracted papers were identified in the current reference list.

**Reviewer #1:**

**Comment 1:**

Thank you for the careful and comprehensive revision of the manuscript. The authors have adequately addressed the comments raised in the previous round of review. The revised manuscript provides clearer methodological justification, appropriate robustness and sensitivity analyses, improved statistical treatment of the repeated-measures data, clearer acknowledgment of the study limitations and scope of inference, and improved data availability. The conclusions are now appropriately framed in relation to the exploratory nature of the study and the use of independent participant groups across stages. I have no further substantive concerns, and I consider the manuscript acceptable for publication in its revised form. This is particularly justified because the authors now explicitly avoid participant-level causal inference when appearance evaluation and behavioral intention were obtained from different participant groups. They also appropriately limit generalizability because purposive sampling was used.

**Response:**

We sincerely thank you for the careful re-evaluation of our manuscript and for the encouraging comments. We are grateful for your recognition of our previous revisions and for the constructive guidance provided throughout the review process.

**Reviewer #2:**

**Comment 1:**

(No Response)

**Response:**

We sincerely thank you for reviewing the revised manuscript. We greatly appreciate your time and evaluation.

**Reviewer #3:**

**Comment 1:**

The author has addressed all of my suggestions; therefore, we believe that the quality of the manuscript has improved and recommend it for publication.

**Response:**

We sincerely thank you for the positive evaluation and for recommending the manuscript for publication. We are grateful for your helpful suggestions and encouraging assessment.

**Reviewer #4:**

**Comment 1:**

The authors have made a substantial effort to address the concerns raised in the previous review round. The revised manuscript demonstrates notable improvements in methodological transparency, statistical rigor, theoretical framing, and discussion of limitations. The justification of the representative-sample selection has been strengthened through additional cluster-validation indices and robustness analyses. The authors have appropriately revised the behavioral-intention analysis using statistical procedures suitable for repeated-measures data and have clarified the interpretation of the entropy-weighted TOPSIS results.

Importantly, the manuscript now more clearly acknowledges key limitations, including the use of purposive sampling, the reliance on standardized two-dimensional stimuli rather than real smart-home devices, and the use of independent participant groups for appearance evaluation and behavioral-intention assessment. The discussion and conclusions have been revised accordingly, avoiding unsupported causal interpretations. Theoretical connections to Kansei engineering, product aesthetics, and age-friendly technology adoption have also been improved.

The study remains exploratory, and some limitations persist, particularly regarding the moderate stability of the clustering solution, the absence of measures of prior technology experience, the use of image-based evaluations, and the lack of confirmatory validation of the extracted appearance dimensions. However, these issues are now explicitly acknowledged and do not undermine the overall contribution of the work.

Overall, the manuscript is technically sound, the analyses are appropriate, the conclusions are generally supported by the presented data, and the authors have adequately addressed the major reviewer concerns. I have no further substantive comments and consider the manuscript suitable for publication.

**Response:**

We sincerely thank you for the thorough and thoughtful re-evaluation of our manuscript. We greatly appreciate your recognition of our previous revisions and the detailed, constructive guidance provided during the review process. We are grateful for your encouraging assessment of the manuscript.

**Reviewer #5:**

**Comment 1:**

The authors have satisfactorily addressed the concerns raised in the previous review. The revised manuscript provides improved clarification of the methodology, experimental setup, results, and supporting discussion. The modifications made in response to the reviewer comments have strengthened the technical presentation and overall quality of the work. The reported results and conclusions are now presented more clearly and consistently, and the manuscript is substantially improved compared with the previous version. I have no further major concerns at this stage. The manuscript may be considered for publication in its revised form, subject to the editor’s final assessment.

**Response:**

We sincerely thank you for the careful re-evaluation of our manuscript and for the encouraging assessment. We are grateful for your constructive feedback and for recognizing the improvements made in the revised manuscript.
